# Supplementary material for: Time-restricted feeding normalizes hyperinsulinemia to inhibit breast cancer in obese postmenopausal mouse models
Source: Nat Commun. 2021 Jan 25;12:565. doi: 10.1038/s41467-020-20743-7 (PMC7835248; doi:10.1038/s41467-020-20743-7)
Supplement: Supplementary file 1 — Supplementary Information [file 41467_2020_20743_MOESM1_ESM.pdf]

Supplementary Information

Title: Time-restricted feeding normalizes hyperinsulinemia to inhibit breast cancer in obese postmenopausal mouse models

Authors: Manasi Das[1,2], Lesley G. Ellies[3,4], Deepak Kumar[1,2], Consuelo Sauceda[1,2], Alexis Oberg[1], Emilie Gross[1,2], Tyler Mandt[5], Isabel G. Newton[5], Mehak Kaur[2], Dorothy D. Sears[2,4,6,7], Nicholas J.G. Webster[1,2,4]*

Affiliations:

[1]VA San Diego Healthcare System, San Diego, CA, USA.

[2]Department of Medicine, Division of Endocrinology and Metabolism, University of California San Diego, La Jolla, CA, USA.

[3]Department of Pathology, University of California San Diego, La Jolla, CA, USA.

[4]Moores Cancer Center, University of California, San Diego, La Jolla, CA, USA.

[5] Department of Radiology, University of California, San Diego, La Jolla, CA, USA.

[6] Department of Family Medicine and Public Health, Division of Preventive Medicine, University of California San Diego, La Jolla, CA, USA.

[7] College of Health Solutions, Arizona State University, Phoenix, AZ, USA

*Address correspondence to: nwebster@ucsd.edu.

Supplementary Figure 1: Changes in metabolic parameters following TRF

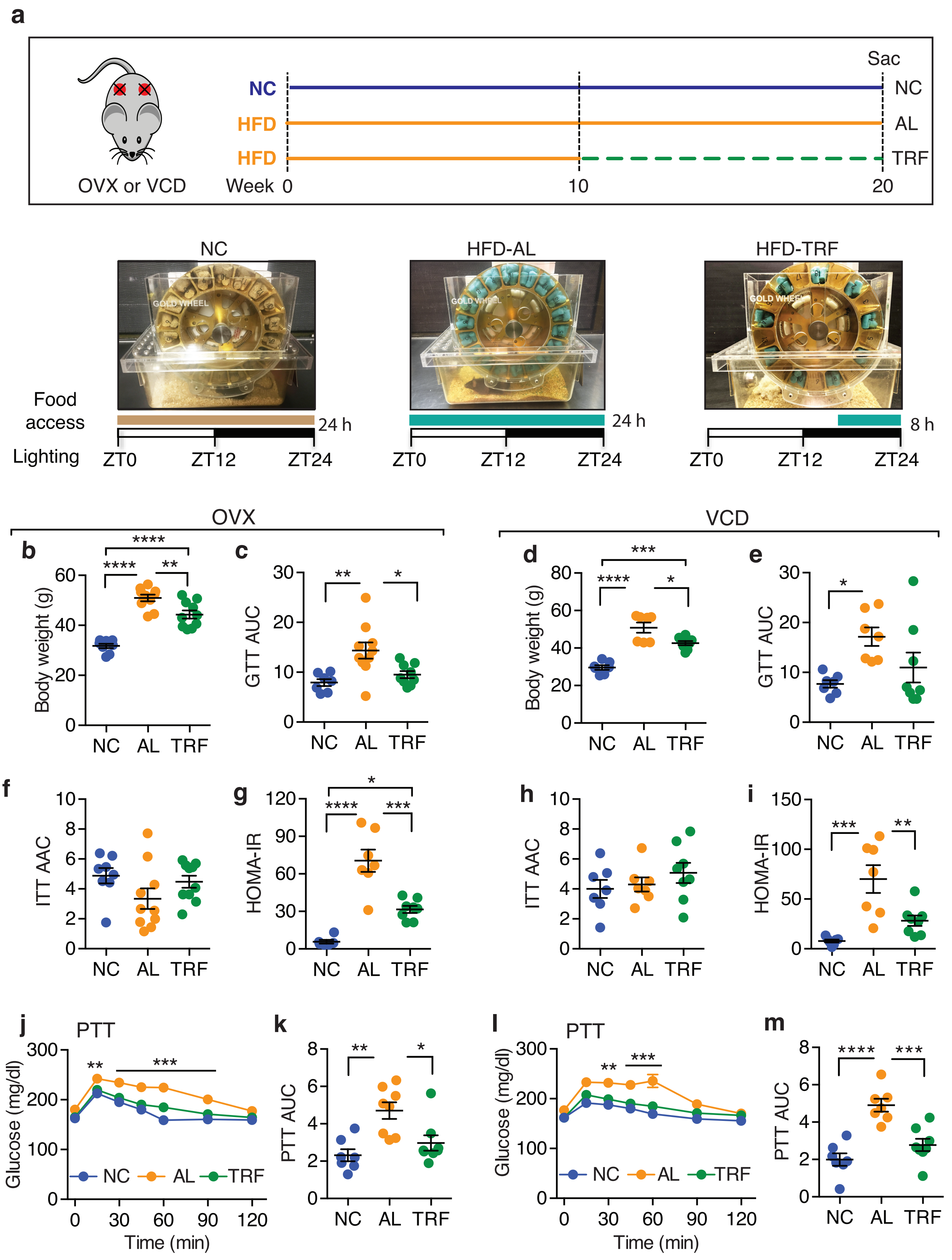

Supplementary Figure 1. **Changes in metabolic parameters following TRF. a)** Schematic representation of the feeding cage and feeding groups such as normal chow *ad libitum* (NC), high fat diet *ad libitum* (AL) and high-fat diet time-restricted feeding (TRF) used in this study. Mice on NC are labeled blue, mice on AL are labeled orange, and mice on TRF are labeled green. Photos show the set-up of the study cages. The food wheel advances at predetermined times to make next aliquot of food available. All groups were housed in the same cages to limit cage effects on nutrition. The TRF cages alternated wells with and without food. The diet, the food access time, and the lighting schedule are shown below the cage photo. ZT0 is lights on at 6 am. The normal chow is beige, the high fat diet is blue. (**b and d**) Terminal body weights of OVX and VCD mice on NC, AL and TRF after 10 weeks of TRF (number of mice n=8 for NC, n=10 for AL, n=10 for NC in panel b; n=7, n=7, n=8 for TRF in panel d; 1-way ANOVA with Tukey multiple comparisons test). (**c and e**) Area under the curve (AUC) for GTT assay in OVX (number of mice n=7 for NC, n=10 for AL, n=9 for TRF) or VCD (n=7 for NC, n=7 for AL, n=8 for TRF) mice following TRF. (**f and h**) Area above the curve (AAC) for ITT assay in OVX (number of mice n=8 for NC, n=10 for AL, n=10 for TRF) or VCD (n=7 for NC, n=7 for AL, n=8 for TRF) mice following TRF. (**g and i**) Homeostatic Model Assessment of Insulin Resistance (HOMA-IR) in OVX (number of mice n=6 for NC, n=7 for AL, n=8 for TRF) and VCD (n=7 for NC, n=7 for AL, n=8 for TRF) mice. (**j and l**) Intraperitoneal-pyruvate tolerance test (PTT) on female OVX (number of mice n=7 for NC, n=8 for AL, n=8 for TRF) or VCD (n=7 for NC, n=7 for AL, n=8 for TRF) mice after 9 weeks of TRF. **(k and m**) Area under the curve (AUC) for PTT assay in OVX (number of mice n=7 for NC, n=8 for AL, n=8 for TRF) or VCD (n=7 for NC, n=7 for AL, n=8 for TRF) mice. Data presented as mean±SEM. For panels b-i, k, and m, data were analyzed by 1-way ANOVA with Tukey's multiple comparison test. For panels j and l, data were analyzed by 2-way ANOVA with Tukey's multiple comparison test. Asterisks show statistical significance as shown. *p<0.05, **p<0.01, ***p<0.001, ****p<0.0001.

Supplementary Figure 2: Time restricted feeding improves liver histology and function.

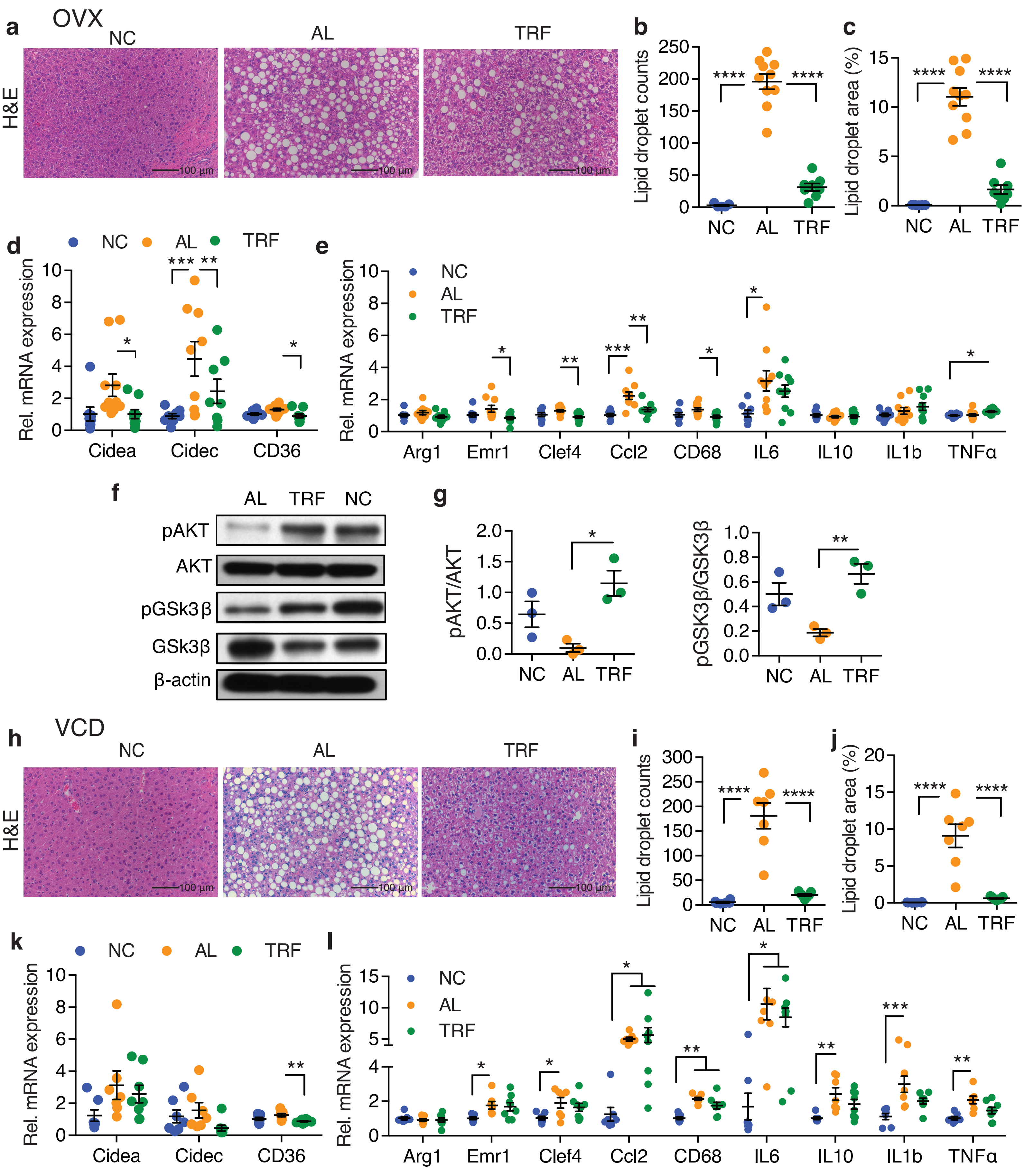

***Supplementary Figure 2*. Time restricted feeding improves liver histology and function. (a and h)** H&E staining of liver sections from OVX and VCD mice on NC, AL and TRF showing hepatic steatosis. Scale bar represents 100 μm. Data shown are representative of two independent experiments. Lipid droplet counts and area measurements in liver from OVX (**b and c**) (number of mice n=5 for NC, n=10 for AL, n=8 for TRF) or VCD (**i and j**) (n=6 for NC, n=7 for AL, n=7 for TRF) mice. (**d and k**) Analysis of lipid storage genes in the liver from OVX (number of mice n=8 for NC, n=10 for AL, n=10 for TRF) or VCD (n=7 for NC, n=7 for AL, n=8 for TRF) mice on NC, AL and TRF. (**e and l**) Analysis of inflammatory genes in the liver from OVX (number of mice n=8 for NC, n=10 for AL, n=10 for TRF) or VCD (n=7 for NC, n=7 for AL, n=8 for TRF) mice on NC, AL and TRF by qPCR. **(f)** Phosphorylation of AKT(Ser473) and GSK3β(Ser9) in liver extracts from NC, AL and TRF OVX mice. Data shown are representative of two independent experiments. **(g)** Quantification of pAKT and pGSK3β phosphorylation normalized to total protein (number of mice n=3/group). Color scheme is the same as Figure S1. Data presented as mean ± SEM. For panels b, c, i, j and g, data were analyzed by 1-way ANOVA with Tukey's multiple comparison test. For panels d, k, e and l, data were analyzed by two-tailed t test. Asterisks show statistical significance as indicated. *$p<0.05$, **$p<0.01$, ***$p<0.001$, ****$p<0.0001$.

Supplementary Figure 3: Time restricted feeding reduces adiposity and inflammation

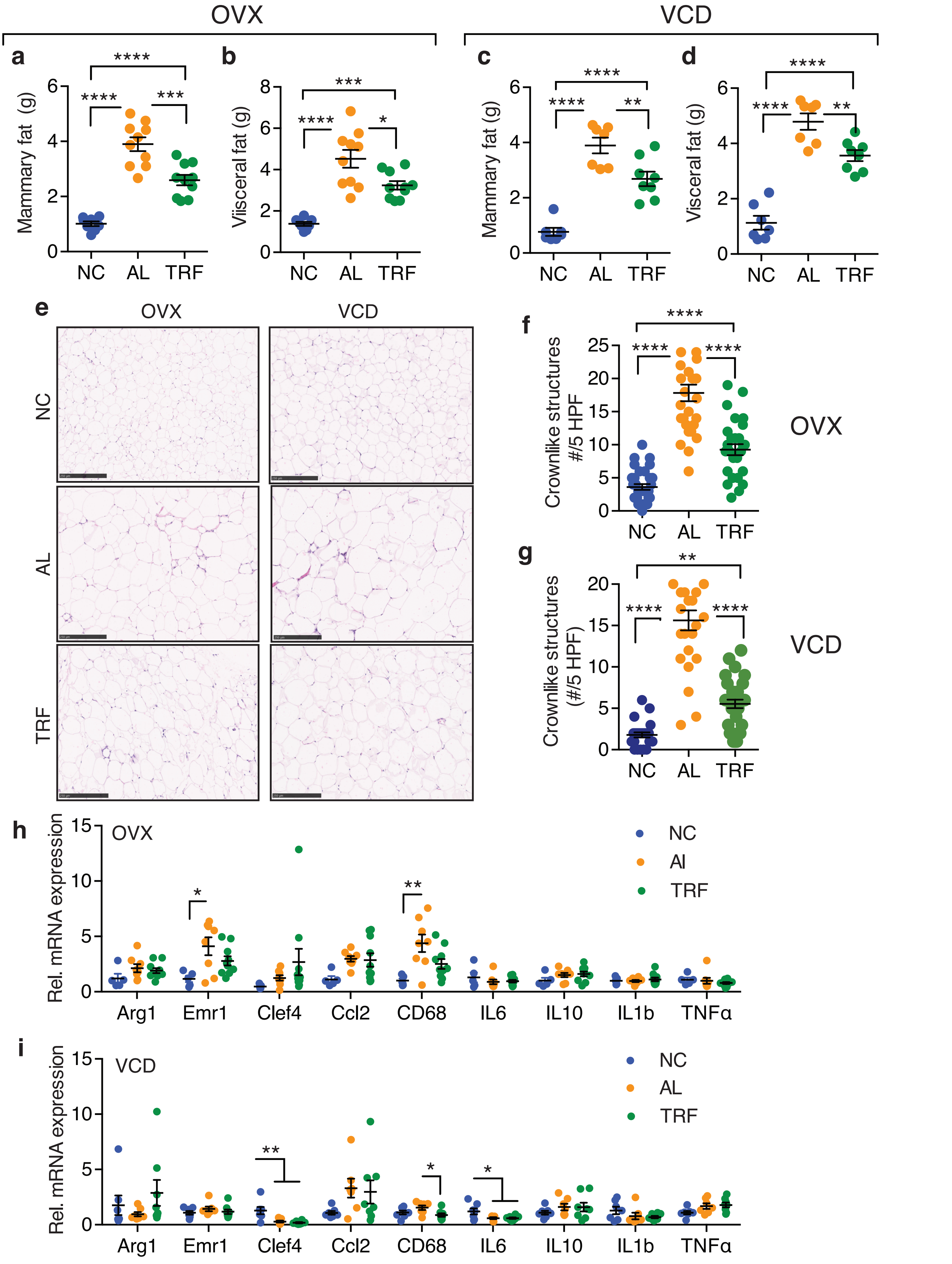

***Supplementary Figure 3*. Time restricted feeding reduces adiposity and inflammation. (a and c)** Mammary fat weight in OVX (number of mice n=8 for NC, n=10 for AL, n=10 for TRF) and VCD (n=7 for NC, n=7 for AL, n=8 for TRF) mice on NC, AL and TRF after euthanasia. (**b and d**) Visceral fat weight in OVX (number of mice n=8 for NC, n=10 for AL, n=10 for TRF) and VCD (n=7 for NC, n=7 for AL, n=8 for TRF) mice on NC, AL and TRF. **(e)** Representative H&E stained sections of MFP showing adipocytes and crown-like structures in OVX and VCD mouse models. Scale bar represents 50 µm. Data shown are representative of two independent experiments. (**f and g**) Quantification of crownlike structure in MFP of OVX (number of tumors n=32 for NC, n=27 for AL, n=28 for TRF), and VCD (n=24 for NC, n=24 for AL, n=32 for TRF) mice. (**h and i**) Analysis of inflammatory gene expression in the mammary fat tissue from OVX (number of mice n=5 for NC, n=8 for AL, n=10 for TRF) or VCD (n=7 for NC, n=7 for AL, n=8 for TRF) mice. Color scheme is the same as Figure S1. Data presented as mean±SEM. For panels a-d, f and g, data were analyzed by 1-way ANOVA with Tukey's multiple comparison test. For panels h and i, data were analyzed by two-tailed t test. Asterisks show statistical significance as indicated. *$p<0.05$, **$p<0.01$, ***$p<0.001$, ****$p<0.0001$.

Supplementary Figure 4: TRF of HFD attenuates tumor growth in obese mice

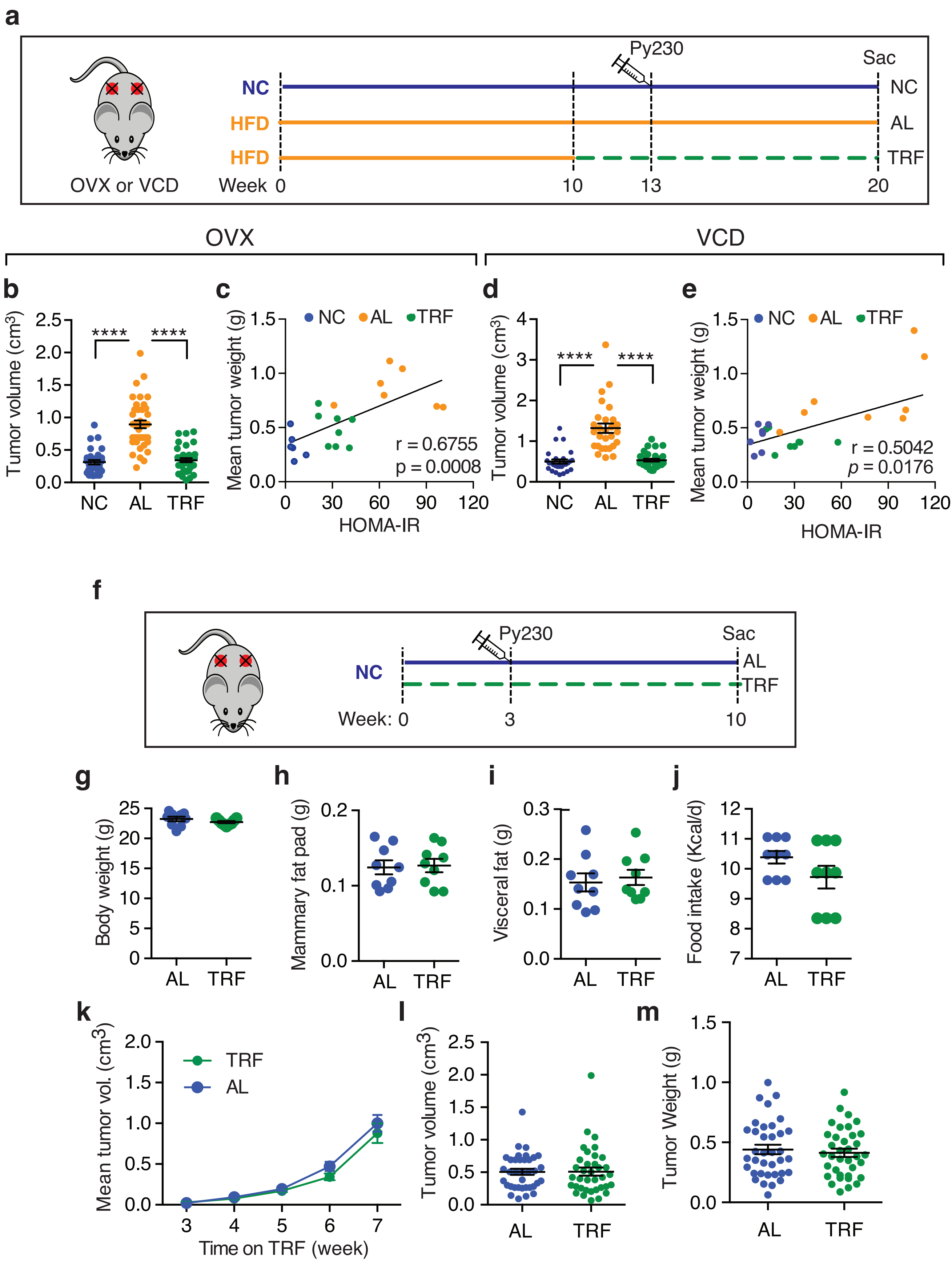

Supplementary Figure 4. **Time restricted feeding of HFD attenuates tumor growth in obese but not lean mice.** **(a)** Schematic showing experimental design with Py230 breast cancer cells. OVX or VCD mice are placed on HFD for 10 weeks to become obese then randomized into AL or TRF groups for a further 10 weeks. Py230 cells were injected 3 weeks after starting TRF. Once tumors were palpable, tumor size was measured weekly with calipers. **(b and d)** Individual tumor volume of OVX (number of tumors n=32 for NC, n=40 for AL, n-40 for TRF) and VCD (n=27 for NC, n=28 for AL, n-32 for TRF) mice on NC, AL, TRF at the end of the study. **(c and e)** Correlation plot of mean tumor weight against HOMA-IR in OVX (number of mice n=6 for NC, n=7 for AL, n=8 for TRF) and VCD (n=7 for NC, n=7 for AL, n=8 for TRF) mice. **(f)** Schematic showing experimental design with Py230 breast cancer cells in lean mice. Lean mice were maintained on *ad libitum* normal chow (NC-AL) or time-restricted normal chow (NC-TRF) for 10 weeks. Py230 cells were injected 3 weeks after starting TRF. Once tumors were palpable, tumor size was measured weekly with calipers. Mice on NC-AL are labeled blue and mice on NC-TRF are labeled green. Body weight **(g),** mammary fat weight **(h),** visceral fat weight **(i)** of OVX mice on NC-AL or NC-TRF at euthanasia, n=9 mice per group. **(j)** Daily food intake of OVX mice on NC-AL or NC-TRF during TRF. **(k)** Mean Py230 tumor volume over time in NC-AL and NC-TRF groups. Individual tumor volume **(l)** and individual tumor weight **(m)** at euthanasia in OVX mice on NC-AL and NC-TRF, n=36 each group. Data presented as mean ± SEM. For panels b, d, g-j, l and m, data were analyzed by two-tailed t test. For panel k, data were analyzed by 2-way ANOVA with Tukey's multiple comparison test. Asterisks show statistical significance TRF vs AL or as indicated; *p<0.05, **p<0.01, ***p<0.001, ****p<0.0001.

Supplementary Figure 5: TRF of HFD attenuates TNBC growth in obese mice

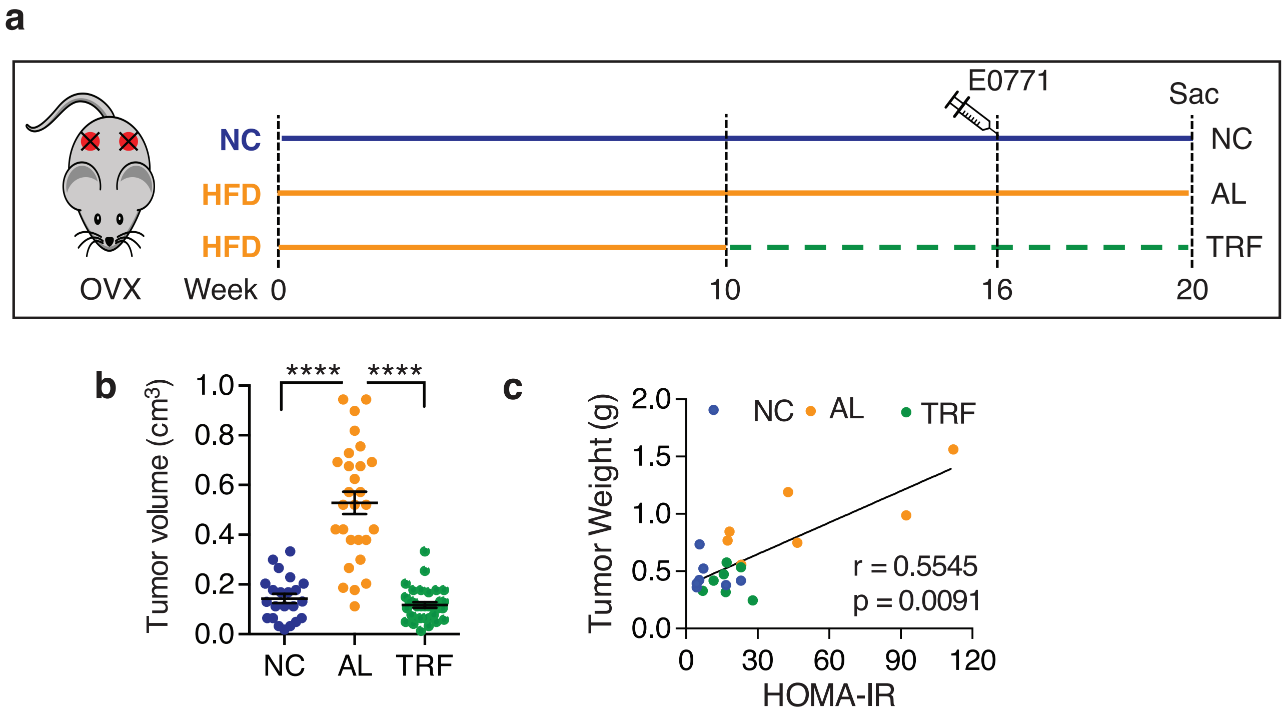

Supplementary Figure 5. **Time restricted feeding of HFD attenuates tumor growth in obese mice.** **(a)** Schematic demonstrating the experimental design using E0771 triple negative breast cancer cells. **(b)** Individual tumor volume after euthanasia for OVX mice on NC, AL, TRF (number of tumors n=22 for NC, n=28 for AL, and n=34 for TRF; analyzed by 1-way ANOVA with Tukey's multiple comparison test). **(c)** Correlation plot of tumor weight v.s. HOMA-IR for OVX mice in NC, AL, TRF groups (n=7 mice per group). Data were analyzed by linear regression using Pearson correlation. Color scheme is the same as Figure S1. Data presented as mean ± SEM. Asterisks show statistical significance TRF vs AL or as indicated; $^{*}p<0.05$, $^{**}p<0.01$, $^{***}p<0.001$, $^{****}p<0.0001$.

Supplementary Figure 6: Py230 and E0771 cells are not sensitive to BHB

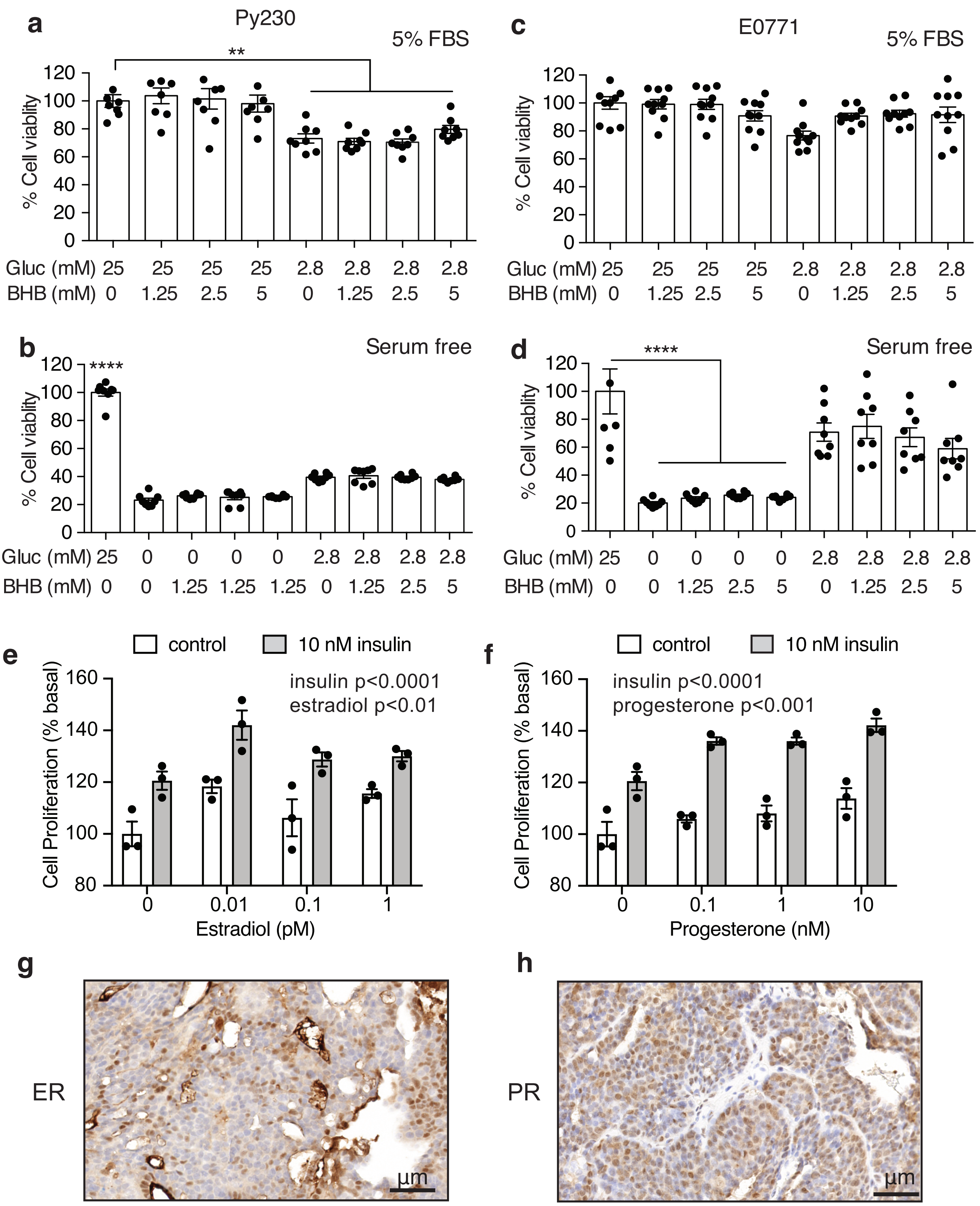

Supplementary Figure 6. **Py230 and E0771 cells are not sensitive to β-hydroxy-butyrate. (a and c)** Py230 cells **(a)** or E0771 cells **(c)** culture in the presence of 5% FBS and the presence of 25 mM or 2.8 mM glucose and increasing concentrations of β-hydroxy-butyrate (BHB, 0-5 mM). (**b and d**) Py230 cells **(b)** or E0771 cells **(d)** culture in the absence of FBS and the presence of 25 mM or 2.8 mM glucose and increasing concentrations of β-hydroxy-butyrate (BHB, 0-5 mM). Cell viability was measured by CellTiter-Glo assay after 24 h. Data presented as mean ± SEM, n=8 replicates. Asterisks indicate statistical significance: **$p<0.01$, ***$p<0.0001$ by 1-way ANOVA. **(e)** Py230 cells were cultured in the presence of increasing concentrations of estradiol (0, 0.01, 0.1, 1 pM) in the presence or absence of 10 nM insulin. Cell number was quantified using a Coulter Counter. Data are expressed as % basal mean ± SEM, n=3 experiments. Two-way ANOVA indicated significant insulin ($p<0.0001$) and estradiol ($p<0.01$) effects but no interaction. **(f)** Py230 cells were cultured in the presence of increasing concentrations of progesterone (0, 0.1, 1, 10 nM) in the presence or absence of 10 nM insulin. Cell number was quantified using a Coulter Counter. Data are expressed as % basal mean ± SEM, n=3 experiments. Two-way ANOVA indicated significant insulin ($p<0.0001$) and progesterone ($p<0.001$) effects but no interaction. (**g**) Py230 tumors (100,000 cells injected) from OVX mice were stained with anti-estrogen receptor α antibody (D12, 1:100 Santa Cruz) following citrate buffer pH6.0 antigen retrieval. Positive nuclei are stained brown (DAB, vector labs) against a counter stain of Meyer's hematoxylin. **(h)** Py230 tumors were stained with anti-progesterone receptor antibody (ab63605, 1:500 Abcam) followed by DAB as above for ER. Data shown are representative of two independent experiments.

Supplementary Figure 7: Fasting glucose levels in mice with insulin pumps or on diazoxide

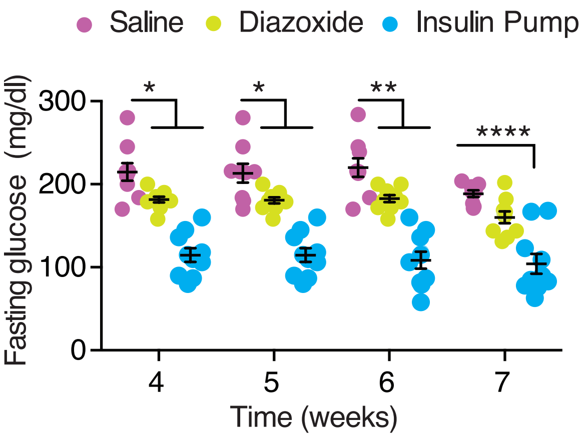

Supplementary Figure 7. **Fasting glucose levels for mice on insulin pumps or diazoxide.** Fasting blood glucose measured every week for mice on HFD implanted with saline or insulin pumps (0.6U/day), or on HFD with diazoxide (1.125 g/kg) (number of mice n=9 for saline, n=10 each for diazoxide and insulin pump). Saline control mice are shown in magenta, insulin pump mice in cyan, and diazoxide mice in yellow. Individual values are shown as well as the mean and SEM in black. Asterisks indicate statistical significance by 2-way ANOVA with Tukey's multiple comparison test; *p<0.05, **p<0.01, ****p<0.0001.

Supplementary Figure 8:TRF enhances endogenous gene circadian rhythms

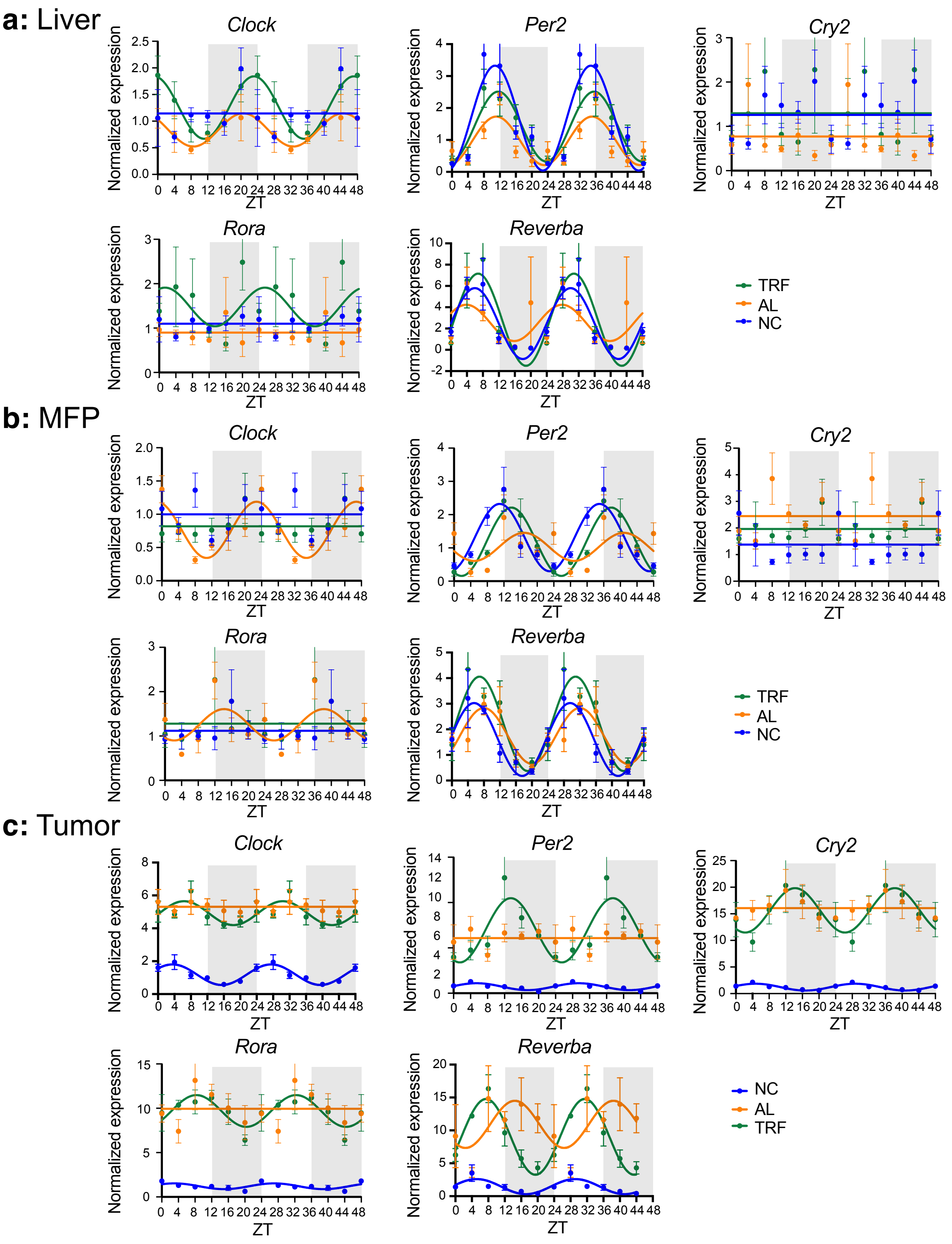

Supplementary Figure 8. **TRF normalizes endogenous gene circadian rhythms in liver, mammary fat and breast tumors. (a, b and c)** QPCR analysis of the clock genes *Clock, Per2, Cry2, Rora* and *Reverba* in liver **(a)** , mammary fat pad (MFP) **(b)** and tumor samples **(c)** collected at different time points (ZT0, 4, 8. 12, 16, 20) over a 24 h period in mice on ad-libitum HFD (AL - orange), time-restricted HFD (TRF - green) or chow diet (NC - blue). ZT0 indicates 6 am and the start of the light phase. Data are presented as mean normalized expression ± SEM (n=4 mice/group). Twenty-four hour rhythms were analyzed statistically using RAIN and circadian curves fitted using PRISM. Genes with significant circadian rhythms are shown as curves, acyclic genes are shown as horizontal lines. Statistical data and circadian parameters are given in Supplemental Table 1. Data are plotted over 48 h to facilitate visualization of the circadian rhythms. Gray rectangles indicate the period of lights off.

Supplementary Figure 9: TRF enhances endogenous protein circadian rhythms in liver

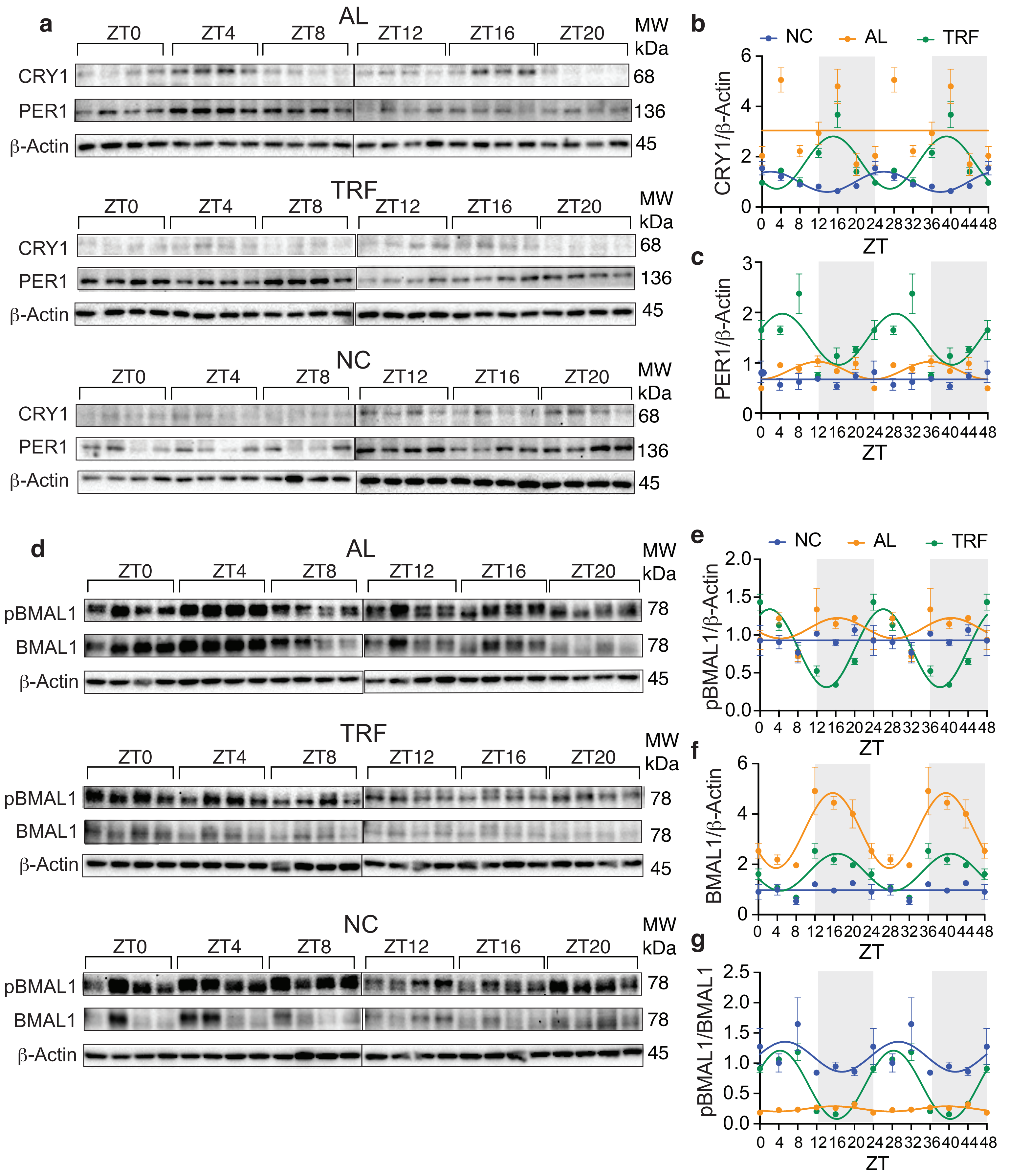

Supplementary Figure 9. **TRF enhances endogenous protein circadian rhythms in liver.** (**a**) Western blot analysis of the clock proteins CRY1 and PER1 in liver samples collected at different time points (ZT0, 4, 8. 12, 16, 20) over a 24 h period in mice on ad-libitum HFD (AL - orange), time-restricted HFD (TRF - green) or chow diet (NC - blue). ZT0 indicates 6 am and the start of the light phase. (**b and c**) Quantification of CRY1 and PER1 protein levels in liver (n=4 mice/group). Normalized expression is graphed over 48 h to facilitate visualization of rhythms. Gray rectangles indicate the period of lights off. (**d**) Western blot analysis of phospho-BMAL1(Ser42) and BMAL1 expression in liver samples collected at different time points (ZT0, 4, 8. 12, 16, 20) over a 24 h period in mice on AL, TRF or NC. **(e to g)** Quantification of the normalized phospho-BMAL1(Ser42), total BMAL1 protein, and phospho-BMAL1/BMAL1 ratio as for b and c. Rhythms over 24 h are analyzed by RAIN and PRISM. Statistical data and circadian parameters are given in Supplemental Table 2. Proteins with significant circadian rhythms are shown as curves, acyclic proteins are shown as horizontal lines. Data are presented as mean expression ± SEM normalized to β-actin (n=4).

Supplementary Figure 10: TRF enhances endogenous protein circadian rhythms in mammary fat pad

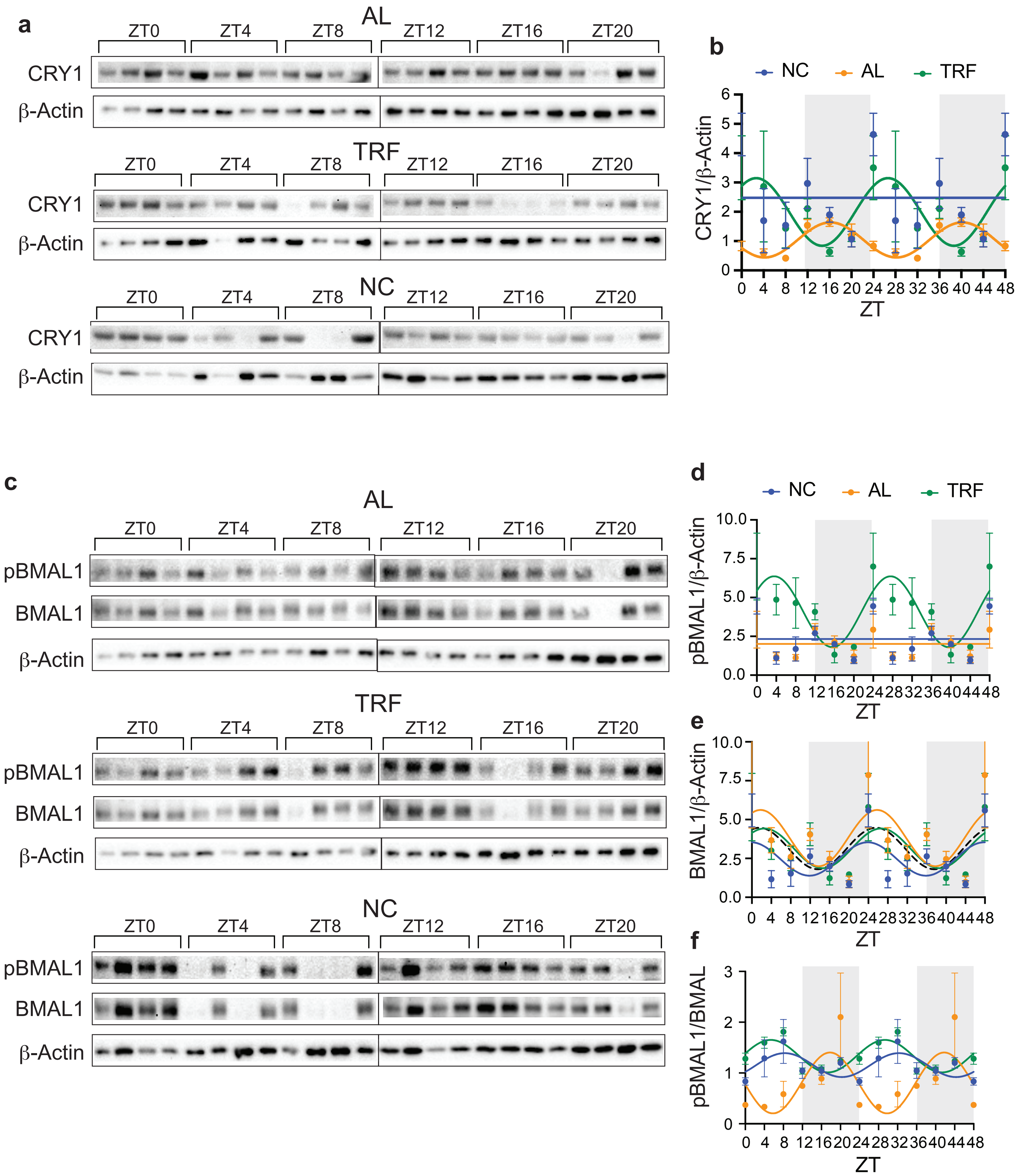

***Supplementary Figure 10*. TRF enhances endogenous protein circadian rhythms in mammary fat pad. (a)** Western blot analysis of the clock protein CRY1 in mammary fat pad samples collected at different time points (ZT0, 4, 8. 12, 16, 20) over a 24 h period in mice on ad-libitum HFD (AL), time-restricted HFD (TRF) or chow diet (NC). PER1 protein was not detectable. ZT0 indicates 6 am and the start of the light phase. **(b)** Quantification of the CRY1 protein expression (AL - orange, TRF – green, NC - blue). Normalized expression is graphed over 48 h to facilitate visualization of rhythms (n=4 mice/group). Gray rectangles indicate the period of lights off. (**c)** Western blot analysis of the phospho-BMAL1(Ser42) and BMAL1 expression in MFP samples collected at different time points (ZT0, 4, 8. 12, 16, 20) over a 24 h period in mice on ad-libitum HFD, TRF or NC. **(d to f)** Quantification of the normalized phospho-BMAL1(Ser42), total BMAL1 protein, and phospho-BMAL1/BMAL1 ratio, respectively, as in b. Rhythms over 24 h are analyzed by RAIN and PRISM. Statistical data and circadian parameters are given in Supplementary Table 2. Proteins with significant circadian rhythms are shown as curves, acyclic proteins are shown as horizontal lines. Data are presented as mean expression ± SEM normalized to β-actin (n=4).

Supplementary Figure 11: Circadian rhythms in Py230 cell in vitro

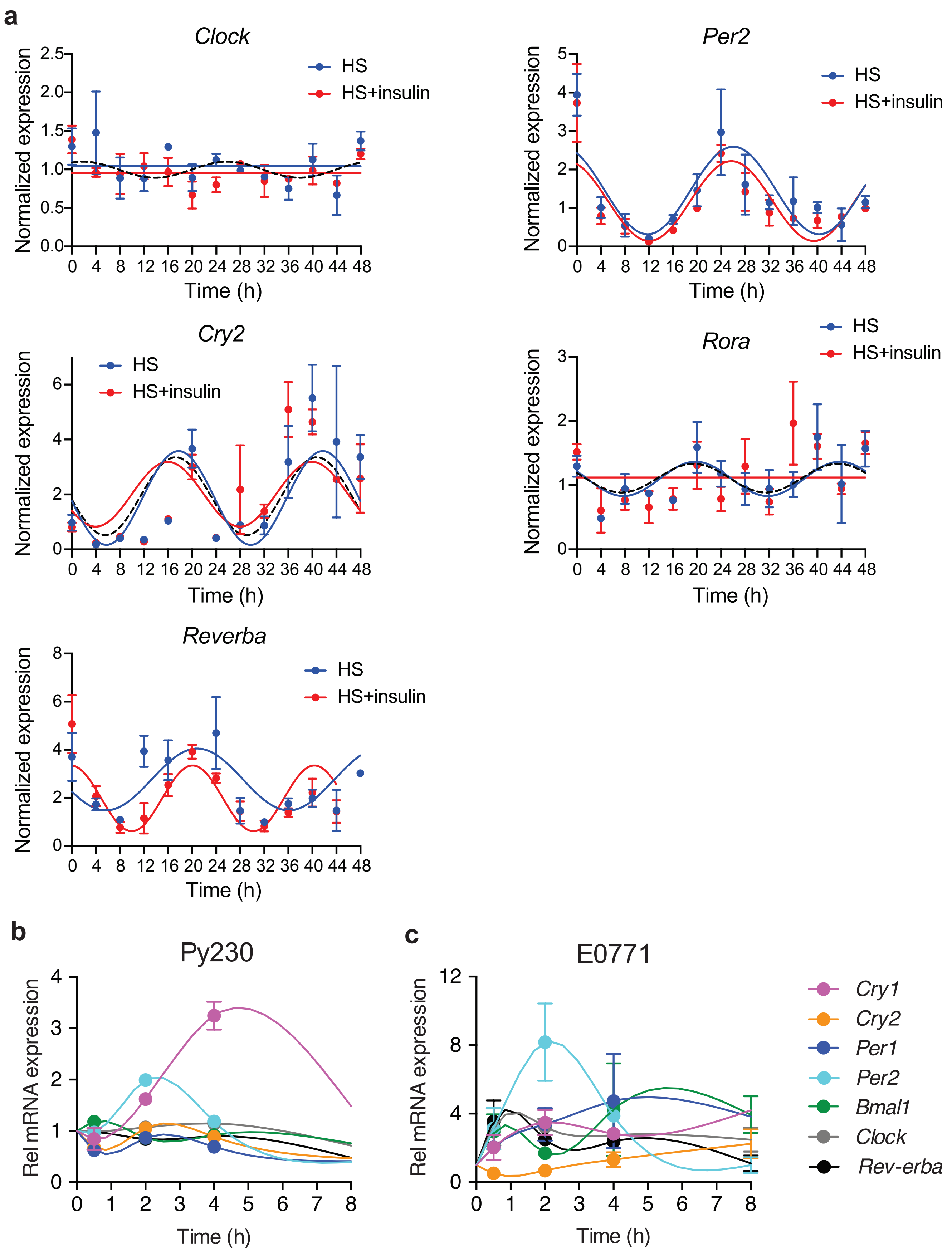

Supplementary Figure 11. **Circadian rhythms in Py230 cell in vitro**. **(a)** QPCR analysis of the clock genes *Clock, Per2*, *Cry2, Rora,* and *Reverba* in Py230 cells following a 50% serum shock without (HS - blue) or with 10 nM insulin (HS+ins – red) collected at different times over 48 h (n=3/time/group). Rhythms over 48 h are analyzed by RAIN and PRISM. Genes with significant circadian rhythms are shown as curves, acyclic genes are shown as horizontal lines. Dotted line indicates a common shared curve that explains the variation in the data. (**b**) Insulin-induced gene expression in Py230 and E0771 cells. Cells were serum starved overnight then treated with 10 nM insulin over 8 h and mRNA expression of clock genes measured by qPCR. Data are presented as mean expression ± SEM (n=3/time). Curve fitting was performed using PRISM8.

Supplementary Table 1: Circadian parameters for gene expression

Circadian analysis of gene expression by RAIN						Prism curve fitting results						
Liver	pVal	phase	peak.shape	period	sig after MTC*		Circadian	single curve fits all	baseline	amplitude	phaseshift	peak (ZT)
Bmal.NC	6.32E-21	24	12	24	Y		Y	N	1.77	2.1	20.1	20
Bmal.AL	1.87E-10	4	8	24	Y		Y		1.46	1.16	19.4	22
Bmal.TRF	1.75E-14	24	16	24	Y		Y		2.16	2.11	19.1	23
Clock.NC	2.56E-03	24	4	24	N		Y	N	1.14	0.33	20.2	18
Clock.AL	5.45E-05	4	8	24	Y		Y		0.84	0.31	19.7	20
Clock.TRF	1.02E-07	4	12	24	Y		Y		1.26	0.58	0.23	24
Per1.NC	5.80E-08	12	12	24	Y		Y	N	1.25	0.59	18.5	8
Per1.AL	8.88E-05	16	12	24	Y		N		0.58			
Per1.TRF	1.48E-02	12	12	24	N		Y		1.49	0.55	7.02	8
Per2.NC	7.09E-17	12	16	24	Y		Y	N	1.68	1.64	3.46	11
Per2.AL	4.98E-08	16	8	24	Y		Y		0.97	0.76	12.8	11
Per2.TRF	1.00E-08	12	20	24	Y		Y		1.42	1.09	22.1	12
Cry1.NC	2.91E-19	24	8	24	Y		Y	N	1.44	1.3	20.7	17
Cry1.AL	4.80E-06	20	16	24	Y		Y		0.83	0.55	16.9	20
Cry1.TRF	4.37E-09	24	12	24	Y		Y		1.27	1.16	19.8	20
Cry2.NC	6.83E-04	24	4	24	Y		Y	Y	1.13	0.12	13.45	8
Cry2.AL	9.59E-03	8	16	24	N		Y					
Cry2.TRF	7.49E-01	24	20	24	N		N					
Rora.NC	5.41E-01	24	8	24	N		N	N	1.09			
Rora.AL	1.07E-02	4	20	24	N		N		0.9			
Rora.TRF	2.80E-07	24	20	24	Y		N		1.48			
Reverba.NC	3.09E-19	8	16	24	Y		Y	N	2.47	3.33	7.86	6
Reverba.AL	6.49E-12	8	16	24	Y		Y		2.53	1.7	5.27	4
Reverba.TRF	2.62E-21	12	12	24	Y		Y		2.83	4.33	7.64	7
MFP	pVal	phase	peak.shape	period	sig after MTC		Circadian	single curve fits all	baseline	amplitude	phaseshift	peak (ZT)
Bmal.NC	4.74E-12	24	12	24	Y		Y	Y	1.59	1.08	13.08	23
Bmal.AL	0.00347573	8	4	24	N		Y					
Bmal.TRF	7.69E-13	24	16	24	Y		Y					
Clock.NC	0.01661444	24	16	24	N		N	N	0.98			
Clock.AL	3.17E-14	4	8	24	Y		Y		0.77	0.42	0.32	23
Clock.TRF	0.17521004	24	8	24	N		Y		0.82	0.19	20.3	18
Per1.NC	0.0010386	4	20	24	Y		N	N	1.38			
Per1.AL	0.16558775	16	16	24	N		Y		1.36	0.49	12.5	12
Per1.TRF	0.00013511	12	12	24	y		Y		1.92	0.74	10.7	8
Per2.NC	1.53E-14	16	12	24	Y		Y	N	1.31	1.01	12.8	11
Per2.AL	2.90E-05	4	4	24	Y		Y		1.03	0.422	11.3	16
Per2.TRF	6.54E-18	16	12	24	Y		Y		1.19	1.03	12.1	14
Cry1.NC	3.59E-18	24	8	24	Y		Y	N	1.37	1.2	10.4	17
Cry1.AL	1.51E-06	16	16	24	Y		Y		1.52	0.69	14.7	16
Cry1.TRF	1.58E-12	24	8	24	Y		Y		1.67	1.06	20.5	18
Cry2.NC	2.71E-05	4	8	24	Y		Y	N	1.32	0.72	0	0
Cry2.AL	0.00211178	12	20	24	N		N		2.49			
Cry2.TRF	0.0623923	24	8	24	N		N		1.95			
Rora.NC	0.75915165	20	8	24	N		Y	Y	1.23	0.33	8.8	14
Rora.AL	3.30E-06	16	16	24	Y		Y					
Rora.TRF	0.15417929	16	16	24	N		Y					
Reverba.NC	3.37E-11	8	16	24	Y		Y	Y	1.6	1.42	4.83	6
Reverba.AL	4.24E-11	12	12	24	Y		Y		1.74	1.09	7.33	8
Reverba.TRF	4.89E-09	12	12	24	Y		Y		2.22	1.84	4.47	7
Tumor	pVal	phase	peak.shape	period	sig after MTC		Circadian	single curve fits all	baseline	amplitude	phaseshift	peak (ZT)
Bmal.NC	0.00076448	4	16	24	Y		Y	N	1.15	0.36	9.2	4
Bmal.AL	0.00246362	12	12	24	N		Y		1.99	0.39	10.55	8
Bmal.TRF	6.94E-07	4	12	24	Y		Y		2.05	0.46	23.75	0
Clock.NC	2.12E-12	8	12	24	Y		Y	N	1.18	0.62	5.36	4
Clock.AL	0.97472968	4	20	24	N		N		5.31			
Clock.TRF	7.74E-06	12	12	24	Y		Y		8.91	0.73	7.76	8
Per1.NC	1.06E-11	8	16	24	Y		Y	N	1.39	0.69	4.89	4
Per1.AL	0.08023191	24	8	24	N		Y		6.62	1.4	11.54	16
Per1.TRF	9.87E-19	16	8	24	Y		Y		12.5	6.24	19.46	10
Per2.NC	1.29E-09	8	16	24	Y		Y	N	1.45	0.76	4.86	4
Per2.AL	0.0949619	24	12	24	N		N		12.14			
Per2.TRF	2.09E-13	16	16	24	Y		Y		13.88	7.08	12.18	12
Cry1.NC	0.22625456	4	20	24	N		N	N	1.15			
Cry1.AL	0.00021714	4	16	24	Y		Y		8.5	1.73	23.2	3
Cry1.TRF	0.06835216	20	8	24	N		Y		8.58	0.87	15.03	14
Cry2.NC	8.03E-10	8	16	24	Y		Y	N	1.22	0.64	5.04	4
Cry2.AL	0.29903718	16	12	24	N		Y		16.26	2.36	12.66	12
Cry2.TRF	5.65E-05	20	12	24	Y		Y		15.66	4.17	11.98	14
Rora.NC	8.17E-08	4	20	24	Y		Y	N	1.21	0.32	23.2	2
Rora.AL	0.02286228	16	16	24	N		Y		10.08	1.87	3.48	10
Rora.TRF	6.72E-07	12	12	24	Y		Y		9.67	1.79	5.24	8
Reverba.NC	1.42E-15	8	16	24	Y		Y	N	1.45	1.14	4.93	5
Reverba.AL	1.23E-05	20	12	24	Y		Y		10.92	3.58	8.78	14
Reverba.TRF	6.06E-14	12	12	24	Y		Y		9.02	5.76	7.47	8

*corr pVal after Bonf should be < 0.002

Supplementary Table 2: Circadian parameters for protein expression

Circadian analysis of protein expression by RAIN						Prism curve fitting results						
Liver	pVal	phase	peak.shape	period	sig		Circadian	single curve fits all	baseline	amplitude	phaseshift	peak (ZT)
Bmal.NC	0.34948917	24	12	24	N		N	N	0.98			
Bmal.AL	4.26E-06	16	20	24	Y		Y		3.34	-1.49	12	16
Bmal.TRF	9.70E-09	16	20	24	Y		Y		1.67	-0.75	11.4	16
pBMAL.NC	0.61728938	16	20	24	N		N	N	0.93			
pBMAL.AL	0.04593712	24	12	24	Y		N		1.1	-0.13	11.3	1
pBMAL.TRF	7.01E-12	4	16	24	Y		Y		0.81	0.49	0	16
pBMAL.ratio	0.44626547	12	4	24	N		N	N	1.11			
pBMSL.ratio.	0.005327	24	4	24	Y		N		0.25			
pBMAL.ratio	2.97E-09	12	8	24	Y		Y		0.64	-0.56	8.3	14
Per1.NC	0.89341372	16	20	24	N		N	N	0.69			
Per1.AL	0.03564365	16	12	24	Y		N		0.94	-0.15	12.7	12
Per1.TRF	0.00035931	12	4	24	Y		Y		1.5	-0.37	8.3	4
Cry1.NC	0.10092606	4	16	24	N		Y	N	0.85			
Cry1.AL	0.10421398	20	4	24	N		N		3.12			
Cry1.TRF	2.30E-06	20	8	24	Y		Y		1.77	1.03	14.9	16
MFP	pVal	phase	peak.shape	period	sig		Circadian	single curve fits all	baseline	amplitude	phaseshift	peak (ZT)
Bmal.NC	0.15605847	4	20	24	N		Y	Y	2.97	1	-0.6	1
Bmal.AL	0.01169886	4	20	24	Y		Y					
Bmal.TRF	0.01826788	4	20	24	Y		Y					
pBMAL.NC	0.45249819	4	20	24	N		N	N	2.28			
pBMAL.AL	0.01965022	16	16	24	Y		N		2.01			
pBMAL.TRF	0.00160175	8	12	24	Y		Y		4.08	2.28	23.1	4
pBMAL.ratio	0.56414672	12	16	24	N		Y	N	1.16	0.24	7.26	8
pBMSL.ratio.	8.91E-06	24	8	24	Y		Y		0.8	0.6	20.5	18
pBMAL.ratio	8.55E-06	12	4	24	Y		Y		1.33	0.32	8.02	5
Cry1.NC	0.77497946	4	20	24	N		N	N	2.43			
Cry1.AL	0.00033236	20	16	24	Y		Y		1.04	0.6	14.6	16
Cry1.TRF	0.09555599	4	16	24	N		Y		1.99	1.15	23.3	2
Tumor	pVal	phase	peak.shape	period	sig		Circadian	single curve fits all	baseline	amplitude	phaseshift	peak (ZT)
Bmal.NC	0.29175637	4	12	24	N		N	N	1.07			
Bmal.AL	0.000257	12	8	24	Y		Y		1.81	0.53	8.19	4
Bmal.TRF	0.1644668	4	16	24	N		Y		1.38	0.29	23	4
pBMAL.NC	0.00081873	12	12	24	Y		Y	N	1.36	0.35	7.55	8
pBMAL.AL	0.000514	12	12	24	Y		Y		2.03	1.26	7.45	8
pBMAL.TRF	0.00282439	8	12	24	Y		Y		1.61	0.58	5.3	4
pBMAL.ratio	9.37E-06	8	20	24	Y		Y	N	1.3	0.3	4.13	8
pBMSL.ratio.	0.00274507	12	16	24	Y		Y		1.12	0.43	7.08	8
pBMAL.ratio	0.00011375	8	8	24	Y		Y		1.2	0.19	5.2	4
Per1.NC	0.0050091	4	4	24	Y		Y	N	0.77	0.12	0.8	20
Per1.AL	0.0426559	24	12	24	Y		Y		1.82	0.29	20.1	20
Per1.TRF	0.11546917	4	12	24	N		Y		1.06	0.13	0.05	0
Cry1.NC	0.5896946	4	20	24	N		N	N	0.91			
Cry1.AL	0.93676615	8	8	24	N		N		0.92			
Cry1.TRF	0.00728343	8	8	24	Y		Y		0.53	0.19	8.6	4

Supplementary Table 3: Name and Sequence of the primers

	Oligonucleotides	
Primer: Cidea	Forward: TGACATTCATGGGATTGCAGAC<br>Reverse: GGCCAGTTGTGATGACTAAGAC	Integrated DNA Technologies
Primer: Cidec	Forward: GATGGACTACGCCATGAAGTC<br>Reverse: GTGCTCACTGCCACATGC	Integrated DNA Technologies
Primer: Cd36	Forward: GGACATTGAGATTCTTTTCCTCTG<br>Reverse: GCAAAGGCATTGGCTGGAAGAAC	Integrated DNA Technologies
Primer: Arg1	Forward: TGGCTTTAACCTTGGCTTGCTTCG<br>Reverse: CATGTGGCGCATTCACAGTCACTT	Integrated DNA Technologies
Primer: Emr1 (F4/80)	Forward: CTTTGGCTATGGGCTTCCAGTC<br>Reverse: GCAAGGAGGACAGAGTTTATCGTG	Integrated DNA Technologies
Primer: Clec4f	Forward: GAGGCCGAGCTGAACAGAG<br>Reverse: TGTGAAGCCACCACAAAAAGAG	Integrated DNA Technologies
Primer: Ccl2	Forward: TTAATGCCCCACTCACCTGC<br>Reverse: GAGCTTGGTGACAAATACTACAGC	Integrated DNA Technologies
Primer: Cd68	Forward: TGTCTGATCTTGCTAGGACCG<br>Reverse: GAGAGTAACGGCCTTTTTGTGA	Integrated DNA Technologies
Primer: IL6	Forward: GCTACCAAACTGGATATAATCAGGA<br>Reverse: CCAGGTAGCTATGGTACTCCAGAA	Integrated DNA Technologies
Primer: IL10	Forward: GCTCTTACTGACTGGCATGAG<br>Reverse: CGCAGCTCTAGGAGCATGTG	Integrated DNA Technologies
Primer: IL1b	Forward: AAGGGCTGCTTCCAAACCTTTGAC<br>Reverse: ATACTGCCTGCCTGAAGCTCTTGT	Integrated DNA Technologies
Primer: TNFα	Forward: TGAGACCAGCCTGTGCTATG<br>Reverse: AAGCCAAAGCTGGTCAGTCTA	Integrated DNA Technologies
Primer: Bmal1	Forward: CCACCTCAGAGCCATTGATACA<br>Reverse: GAGCAGGTTTAGTTCCACTTTGTCT	Integrated DNA Technologies
Primer: Clock	Forward: ACCATGACGGCCCCACAAGC<br>Reverse: GCCACAGAACAGTACCCAGAGTGC	Integrated DNA Technologies
Primer: Per1	Forward: GAGCTACTGCTCCAAGAAGACTCTC<br>Reverse: GTACTTGCTTGTATGGCTGCTCTGAC	Integrated DNA Technologies
Primer: Per2	Forward: AAAGTATTTGCTGGTGTGACTTG<br>Reverse: CAAAGGCACCTCCAACATG	Integrated DNA Technologies
Primer: Cry1	Forward: ATAGACGCAGCGGATGGTGTCG<br>Reverse: AGCGCAGGTGTCGGTTATGAGC	Integrated DNA Technologies
Primer: Cry2	Forward: ACAGCAAGCCTCTCCTCCT<br>Reverse: GAGATGCCACCCTCTGTAGC	Integrated DNA Technologies
Primer: Rev-erba	Forward: GTCTCTCCGTTGGCATGTCT<br>Reverse: CCAAGTTCATGGCGCTCT	Integrated DNA Technologies
Primer: Rora	Forward: ACGCCCACCTACAACATCTC<br>Reverse: TGCCCATCCATATAGGTCCT	Integrated DNA Technologies
Primer: M36b4	Forward: ACCCTGAAGTGCTCGACATCACAG<br>Reverse: GCAGGGGCAGCAGCCGCAAATGC	Integrated DNA Technologies
Primer: Ppia	Forward: GGCAAATGCTGGACCAAAC<br>Reverse: CATTCCTGGACCCAAAACG	Integrated DNA Technologies
