# Supplementary material for: Time-restricted feeding normalizes hyperinsulinemia to inhibit breast cancer in obese postmenopausal mouse models
Source: Nat Commun. 2021 Jan 25;12:565. doi: 10.1038/s41467-020-20743-7 (PMC7835248; doi:10.1038/s41467-020-20743-7)
Supplement: Supplementary file 3 — Reporting Summary [file 41467_2020_20743_MOESM3_ESM.pdf]

nature research

Corresponding author(s): Nicholas Webster

Last updated by author(s): Nov 10, 2020

Reporting Summary

Nature Research wishes to improve the reproducibility of the work that we publish. This form provides structure for consistency and transparency in reporting. For further information on Nature Research policies, see our Editorial Policies and the Editorial Policy Checklist.

Statistics

For all statistical analyses, confirm that the following items are present in the figure legend, table legend, main text, or Methods section.

n/a	Confirmed	
[ ]	[x]	The exact sample size (*n*) for each experimental group/condition, given as a discrete number and unit of measurement
[ ]	[x]	A statement on whether measurements were taken from distinct samples or whether the same sample was measured repeatedly
[ ]	[x]	The statistical test(s) used AND whether they are one- or two-sided<br>*Only common tests should be described solely by name; describe more complex techniques in the Methods section.*
[x]	[ ]	A description of all covariates tested
[ ]	[x]	A description of any assumptions or corrections, such as tests of normality and adjustment for multiple comparisons
[ ]	[x]	A full description of the statistical parameters including central tendency (e.g. means) or other basic estimates (e.g. regression coefficient) AND variation (e.g. standard deviation) or associated estimates of uncertainty (e.g. confidence intervals)
[ ]	[x]	For null hypothesis testing, the test statistic (e.g. *F*, *t*, *r*) with confidence intervals, effect sizes, degrees of freedom and *P* value noted<br>*Give P values as exact values whenever suitable.*
[x]	[ ]	For Bayesian analysis, information on the choice of priors and Markov chain Monte Carlo settings
[x]	[ ]	For hierarchical and complex designs, identification of the appropriate level for tests and full reporting of outcomes
[x]	[ ]	Estimates of effect sizes (e.g. Cohen's *d*, Pearson's *r*), indicating how they were calculated

Our web collection on statistics for biologists contains articles on many of the points above.

Software and code

Policy information about availability of computer code

Data collection	N/A
Data analysis	N/A

For manuscripts utilizing custom algorithms or software that are central to the research but not yet described in published literature, software must be made available to editors and reviewers. We strongly encourage code deposition in a community repository (e.g. GitHub). See the Nature Research guidelines for submitting code & software for further information.

Data

Policy information about availability of data

All manuscripts must include a data availability statement. This statement should provide the following information, where applicable:

- Accession codes, unique identifiers, or web links for publicly available datasets
- A list of figures that have associated raw data
- A description of any restrictions on data availability

The data that support the findings of this study are available from the corresponding author upon reasonable request.

Field-specific reporting

Life sciences study design

All studies must disclose on these points even when the disclosure is negative.

Sample size	Sample size for tumor growth studies was determined from power calculations based on preliminary data.
Data exclusions	Mice were required to be >40g to be considered obese and included in the study. This is a pre-established condition that we always use. QPCR data that were >2 SD from the mean were flagged as outliers but were only excluded is the Ct values were >35 which is considered the highest reliable value for sybrgreen assays.
Replication	Tumor growth experiments have been repeated in multiple cohorts on different occasions, with different lots of tumor cells.
Randomization	Mice were randomized to the diet groups so that the initial mean body weights were the same.
Blinding	Semi-quantitative assessment of histology was performed by a pathologist who was blinded as to the identity of the samples.

Reporting for specific materials, systems and methods

We require information from authors about some types of materials, experimental systems and methods used in many studies. Here, indicate whether each material, system or method listed is relevant to your study. If you are not sure if a list item applies to your research, read the appropriate section before selecting a response.

Materials & experimental systems

n/a	Involved in the study	
☐	☒	Antibodies
☐	☒	Eukaryotic cell lines
☒	☐	Palaeontology and archaeology
☐	☒	Animals and other organisms
☒	☐	Human research participants
☒	☐	Clinical data
☒	☐	Dual use research of concern

Methods

n/a	Involved in the study	
☒	☐	ChIP-seq
☒	☐	Flow cytometry
☒	☐	MRI-based neuroimaging

Antibodies

Antibodies used	Antibody to PER1 (ab136451), CRY1 (ab104736), BMAL1 (ab 93806), rabbit polyclonal anti-Ki67 antibody (15580), rabbit polyclonal anti-CD31 antibody (28364),were purchased from Abcam (Cambridge, MA). Anti-phospho-AKT (Thr308, 4056), anti-phospho-AKT (Ser473, 9271), anti-panAKT (9272), anti-phospho-GSK-3β (Ser9, 9336), anti-GSK-3β (9315), anti-β actin (3700) antibodies, anti-phospho-ERK1/2 (Thr202/Tyr204, 4377), anti-ERK1/2 (4695) and phospho-BMAL1 (Ser42, 13936) were purchased from Cell Signaling Technology (Danvers, MA).
Validation	Abs were used according to manufacturers protocols and manufacturers validation.

Eukaryotic cell lines

Policy information about cell lines

Cell line source(s)	Py230 cells were generated by Dr. Ellies. E0771 cells were purchased from CH3BioSystem, Amherst, NY.
Authentication	Py230 growth characteristics were routinely checked before a tumor injection study as cells lose their phenotype on prolonged culture, in particular they lose ER and PR expression. Cells available from ATCC are late passage and unrelaible. E0771 cells were authenticated by the manufacturer. Vials were frozen immediately on arrival and fresh vials used for tumor experiments each time.
Mycoplasma contamination	We routinely checked all our cell lines for mycoplasma using a PCR assay.
Commonly misidentified lines (See ICLAC register)	None

Animals and other organisms

Policy information about studies involving animals; ARRIVE guidelines recommended for reporting animal research

Laboratory animals	C57BL/6J mice
Wild animals	None

Field-collected samples	None
Ethics oversight	All animal studies were approved by the UCSD IACUC.

Note that full information on the approval of the study protocol must also be provided in the manuscript.
