# Supplementary material for: Time-restricted feeding normalizes hyperinsulinemia to inhibit breast cancer in obese postmenopausal mouse models
Source: Nat Commun. 2021 Jan 25;12:565. doi: 10.1038/s41467-020-20743-7 (PMC7835248; doi:10.1038/s41467-020-20743-7)
Supplement: Supplementary file 4 — Source Data [file 41467_2020_20743_MOESM4_ESM.pdf]

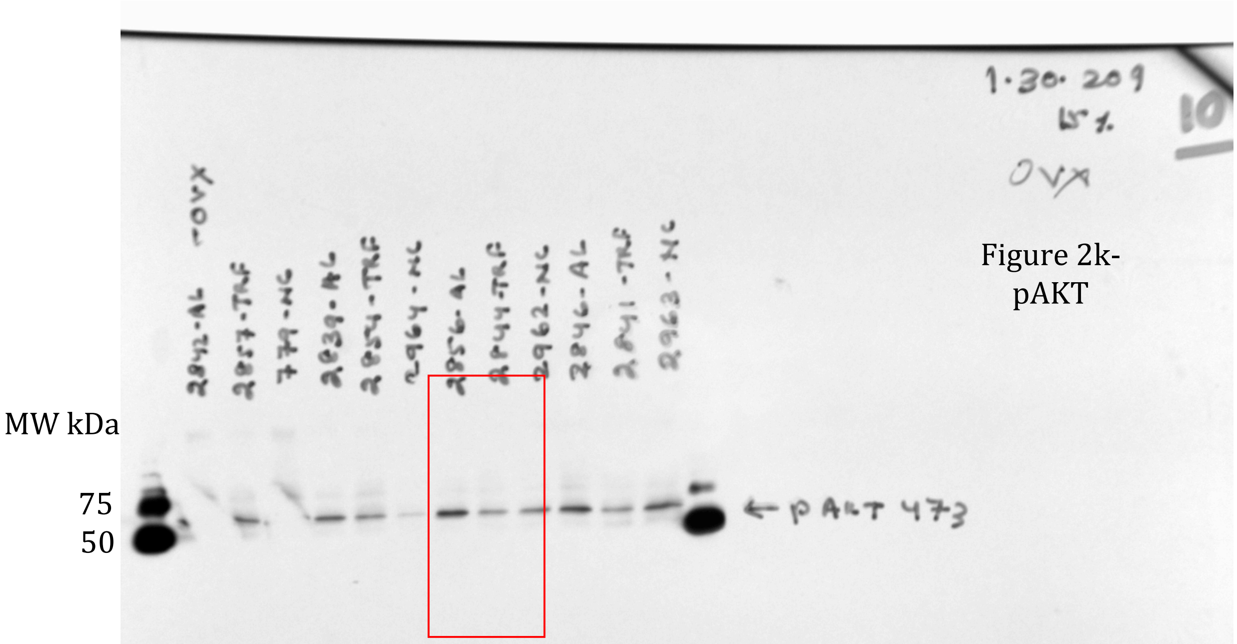

Figure 2k-
pAKT
MW kDa
75
50
1-30-2019
15%
OVX
2842-AL
2857-TRF
779-NC
2839-AL
2854-TRF
2964-NC
2856-AL
2844-TRF
2962-NC
2846-AL
2841-TRF
2963-NC
← pAKT 473

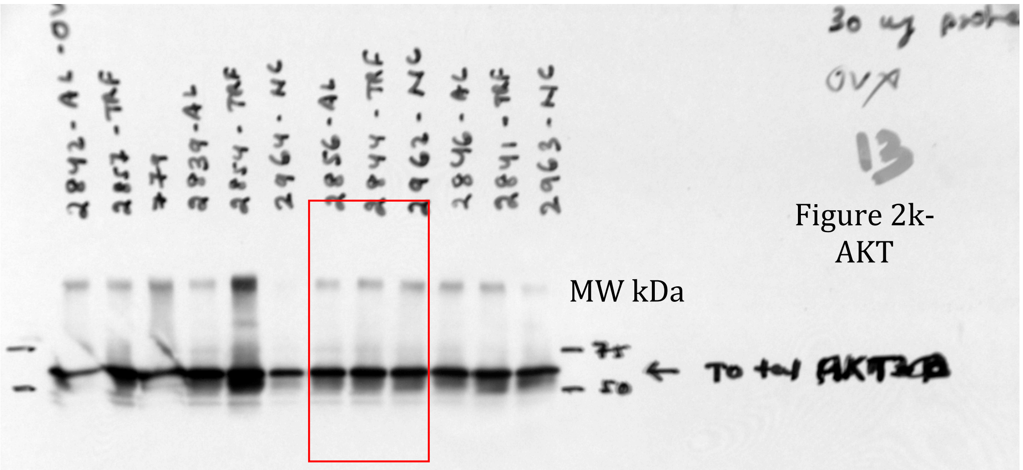

Figure 2k-
AKT
MW kDa
OVX
13
2842-AL
2857-TRF
2839-AL
2854-TRF
2964-NC
2856-AL
2844-TRF
2962-NC
2846-AL
2841-TRF
2963-NC
75
50

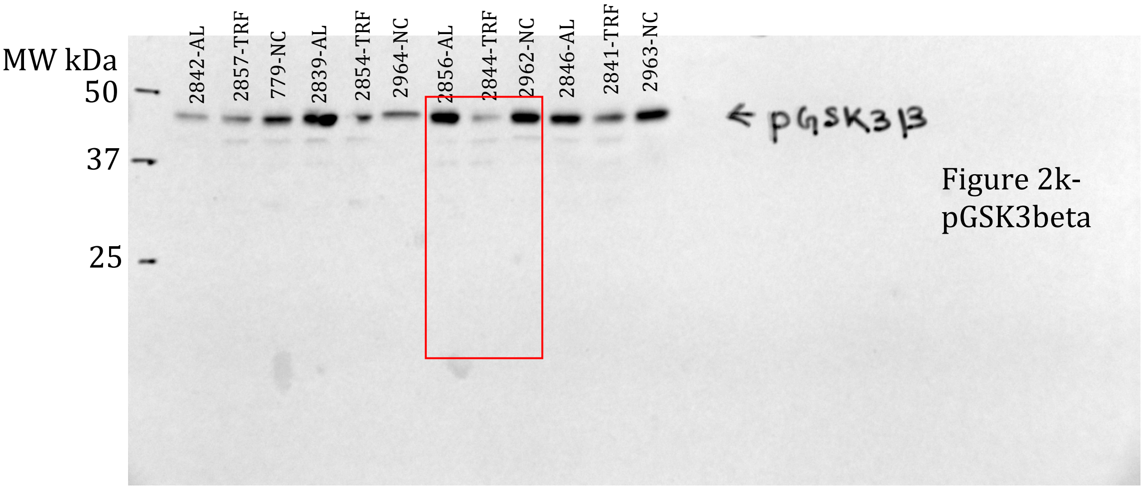

MW kDa
50
37
25
2842-AL
2857-TRF
779-NC
2839-AL
2854-TRF
2964-NC
2856-AL
2844-TRF
2962-NC
2846-AL
2841-TRF
2963-NC
← pGSK3β
Figure 2k-
pGSK3beta

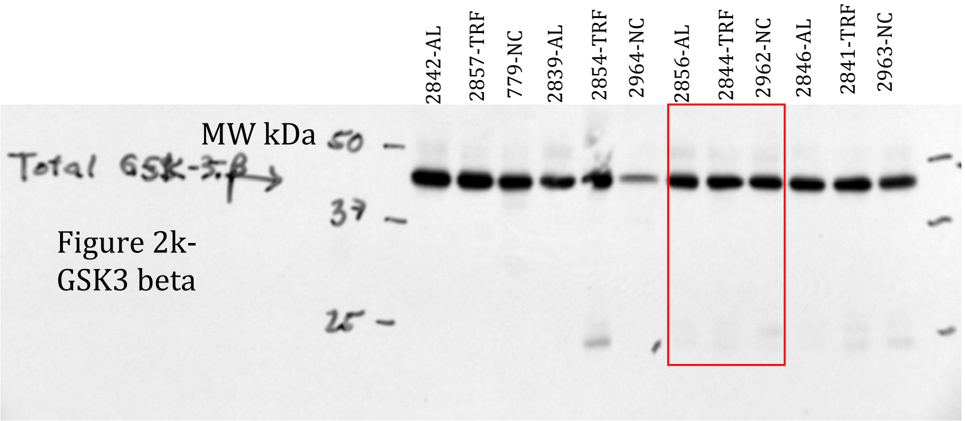
2842-AL
2857-TRF
779-NC
2839-AL
2854-TRF
2964-NC
2856-AL
2844-TRF
2962-NC
2846-AL
2841-TRF
2963-NC
MW kDa
50
37
25
Total GSK-3β
Figure 2k-
GSK3 beta

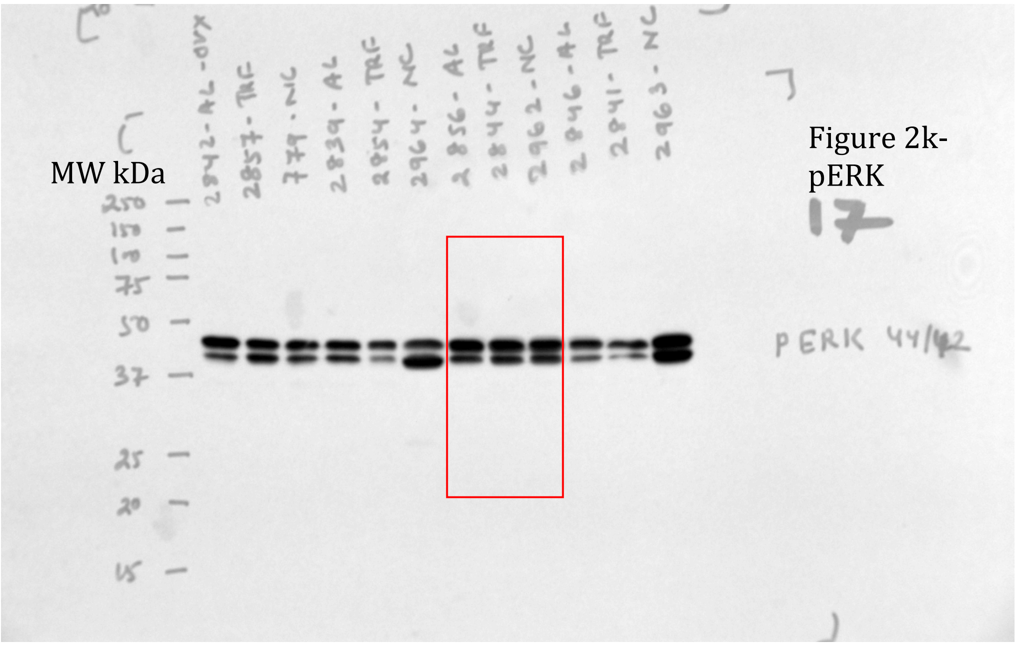
MW kDa
250
150
100
75
50
37
25
20
15
Figure 2k-
pERK
pERK 44/42

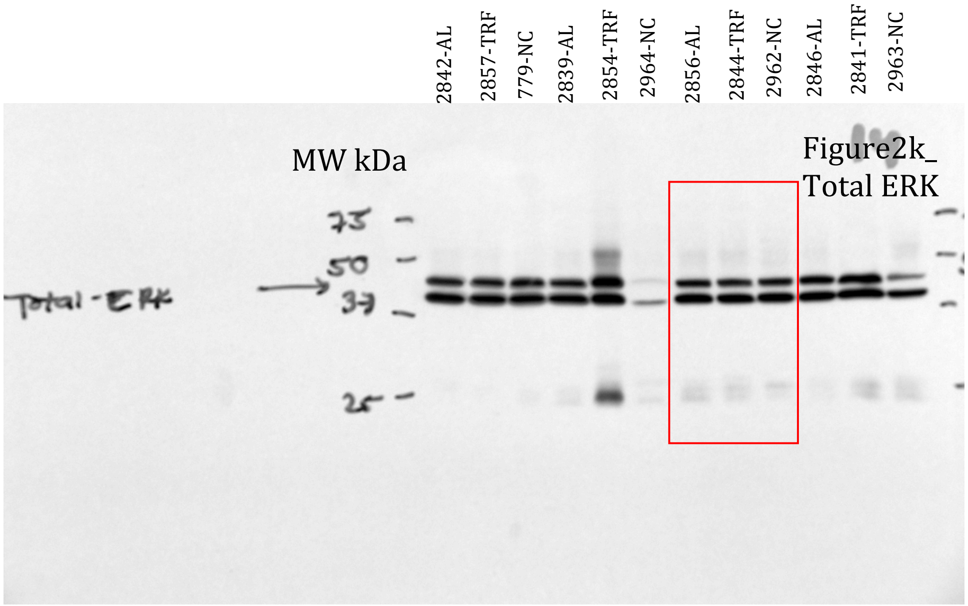
2842-AL
2857-TRF
779-NC
2839-AL
2854-TRF
2964-NC
2856-AL
2844-TRF
2962-NC
2846-AL
2841-TRF
2963-NC
MW kDa
75
50
37
25
Total-ERK
Figure2k_
Total ERK

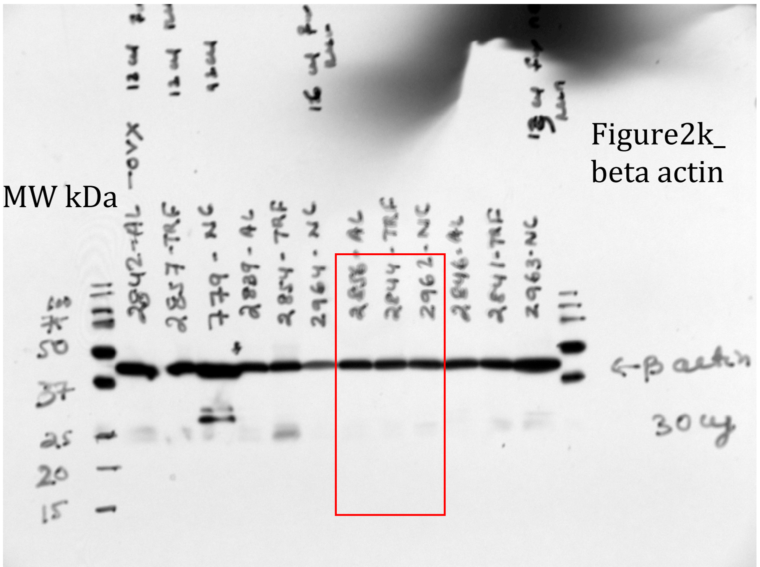
MW kDa
Figure2k_
beta actin
50
37
25
20
15
← β actin
30ug

MW
kDa

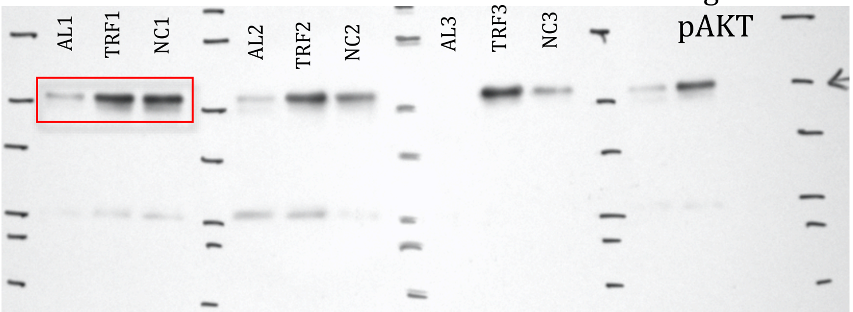
FigureS2f
pAKT
75
50
37
25
20
AL1
TRF1
NC1
AL2
TRF2
NC2
AL3
TRF3
NC3

MW
kDa

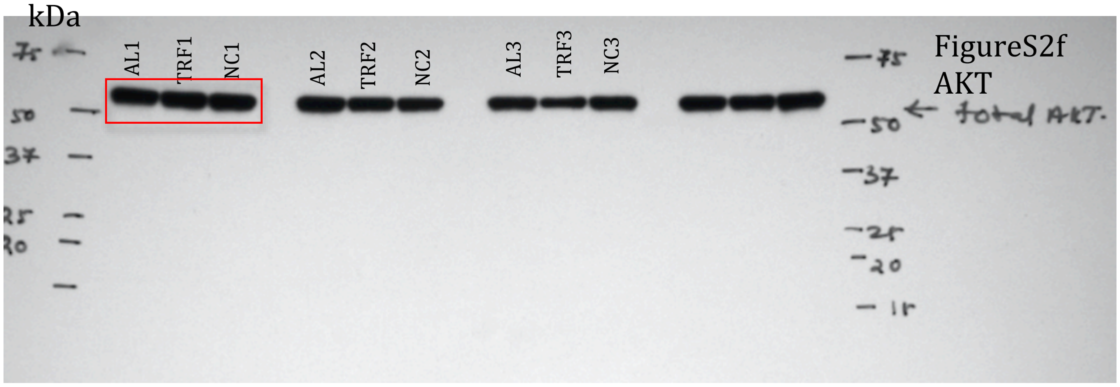
FigureS2f
AKT
AL1
TRF1
NC1
AL2
TRF2
NC2
AL3
TRF3
NC3
75
50
37
25
20
← total AKT.

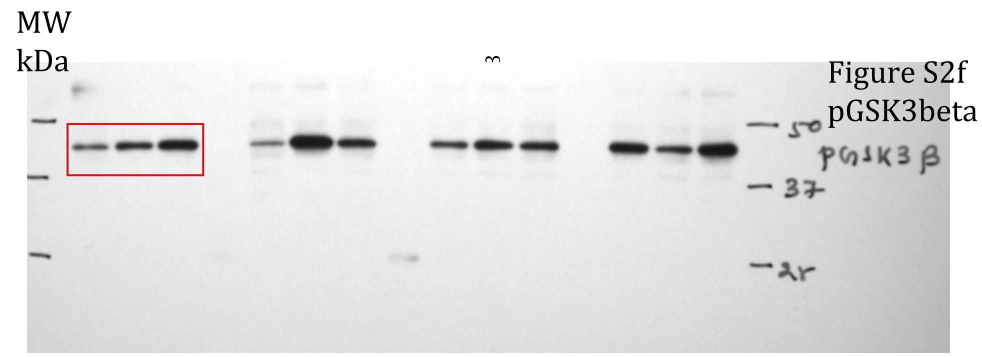
MW
kDa
Figure S2f
pGSK3beta
50
pGSK3β
37
25

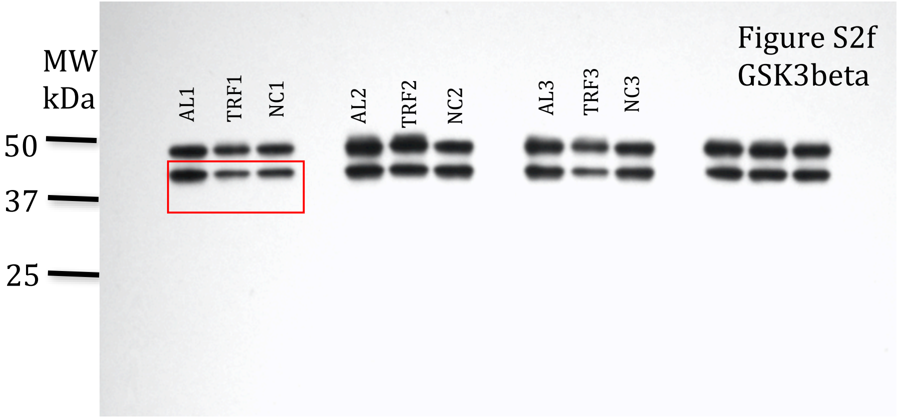
MW
kDa
Figure S2f
GSK3beta
AL1
TRF1
NC1
AL2
TRF2
NC2
AL3
TRF3
NC3
50
37
25

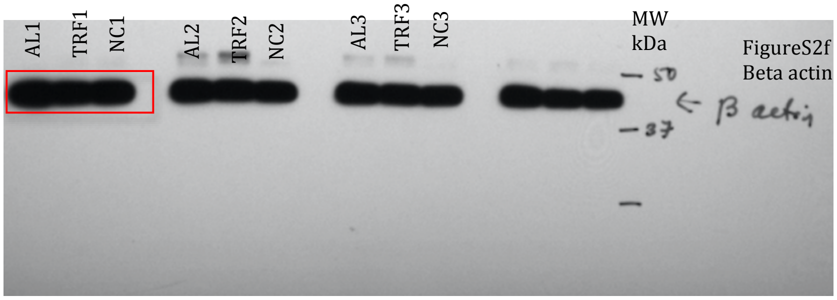
AL1
TRF1
NC1
AL2
TRF2
NC2
AL3
TRF3
NC3
MW
kDa
FigureS2f
Beta actin
50
37
β actin

Figure 9a

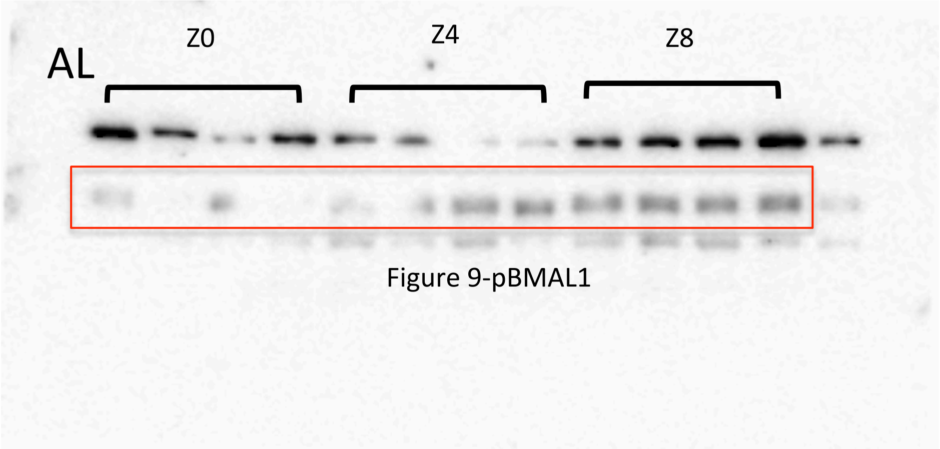

Figure 9-pBMAL1

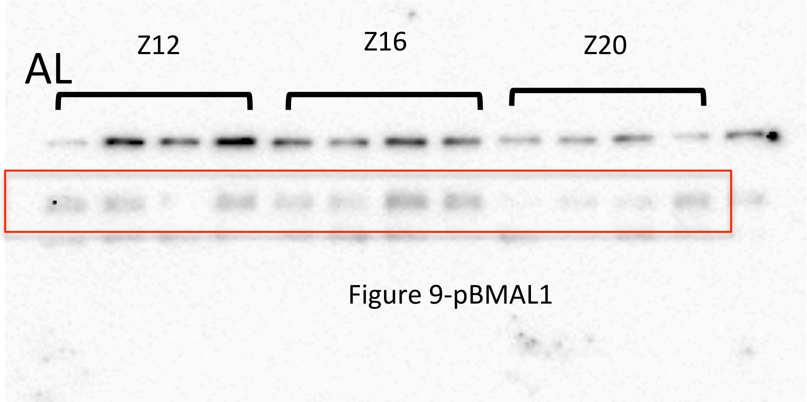

Figure 9-pBMAL1

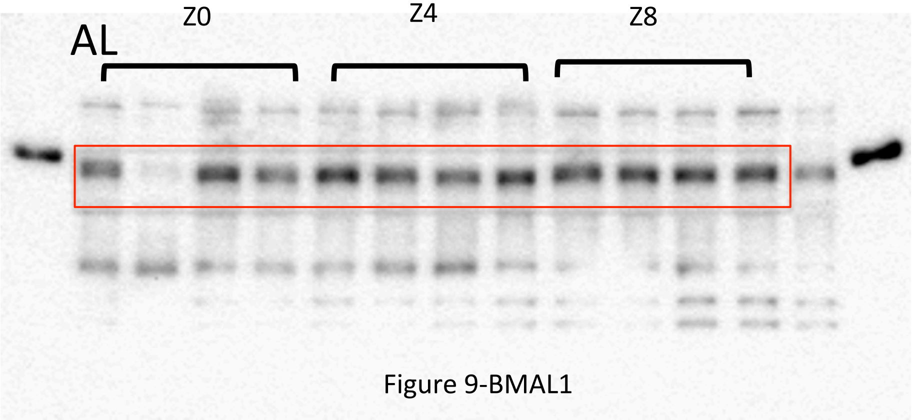

Figure 9-BMAL1

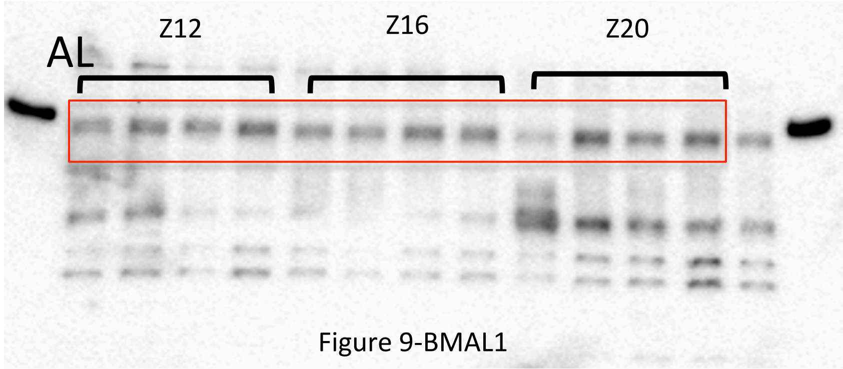

Figure 9-BMAL1

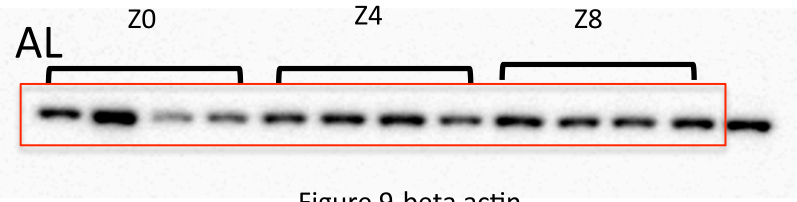

Figure 9-beta actin

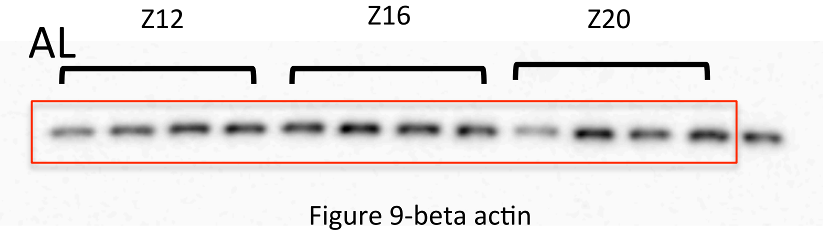

Figure 9-beta actin

Figure 9a

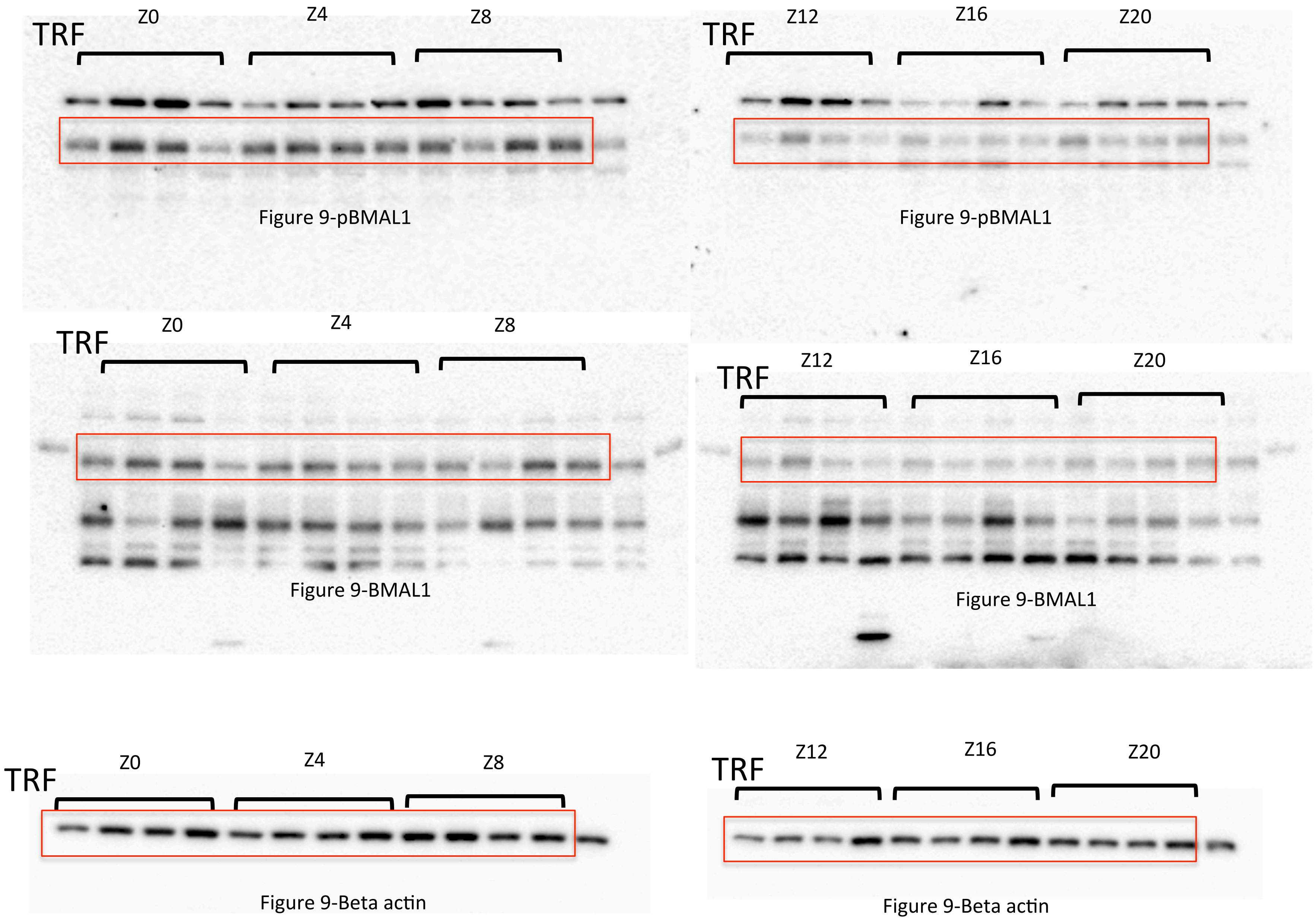
TRF
Z0
Z4
Z8
Figure 9-pBMAL1
TRF
Z12
Z16
Z20
Figure 9-pBMAL1
TRF
Z0
Z4
Z8
Figure 9-BMAL1
TRF
Z12
Z16
Z20
Figure 9-BMAL1
TRF
Z0
Z4
Z8
Figure 9-Beta actin
TRF
Z12
Z16
Z20
Figure 9-Beta actin

Figure 9a

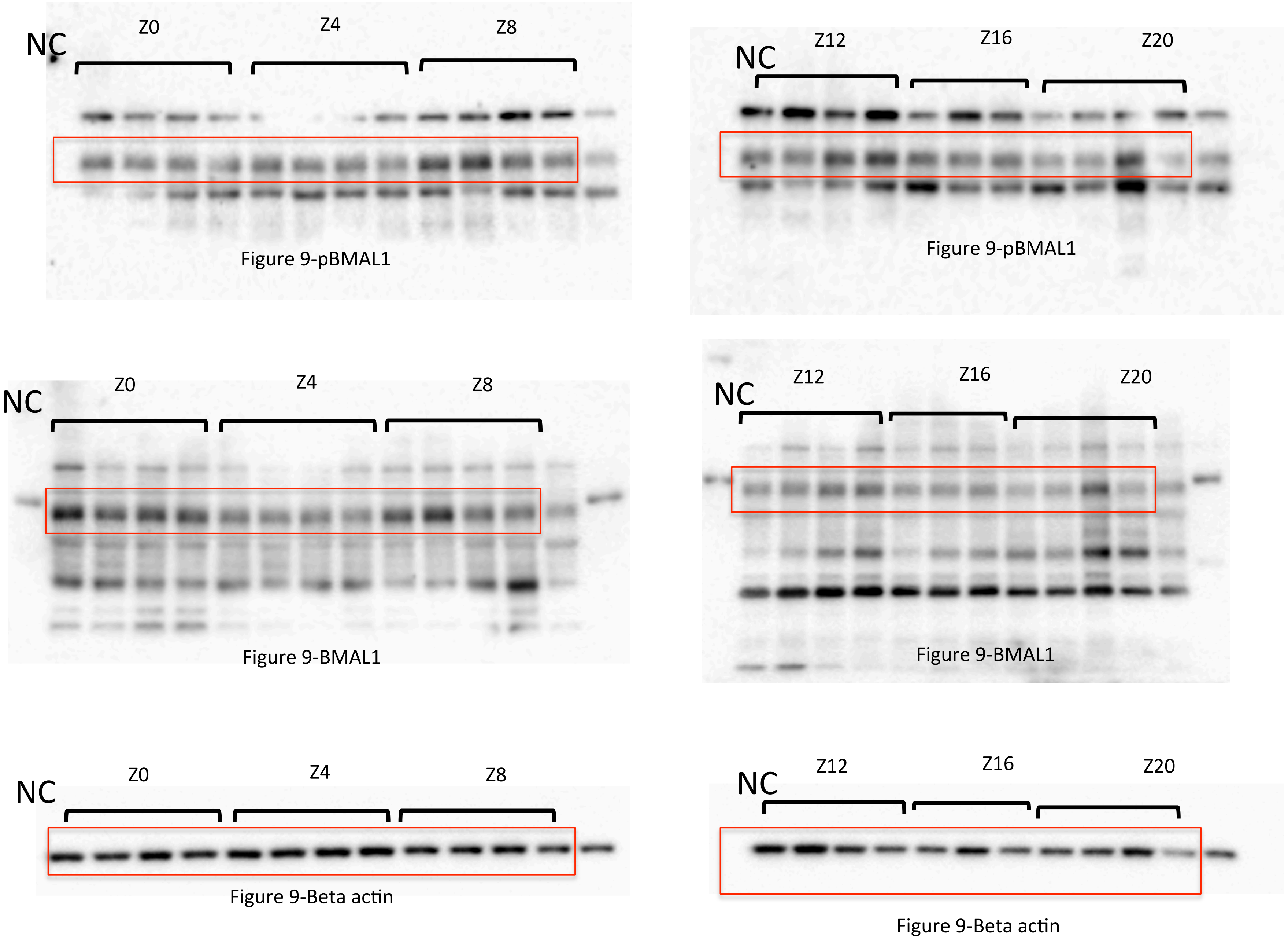
NC
Z0
Z4
Z8
Figure 9-pBMAL1
NC
Z12
Z16
Z20
Figure 9-pBMAL1
NC
Z0
Z4
Z8
Figure 9-BMAL1
NC
Z12
Z16
Z20
Figure 9-BMAL1
NC
Z0
Z4
Z8
Figure 9-Beta actin
NC
Z12
Z16
Z20
Figure 9-Beta actin

Figure 9c

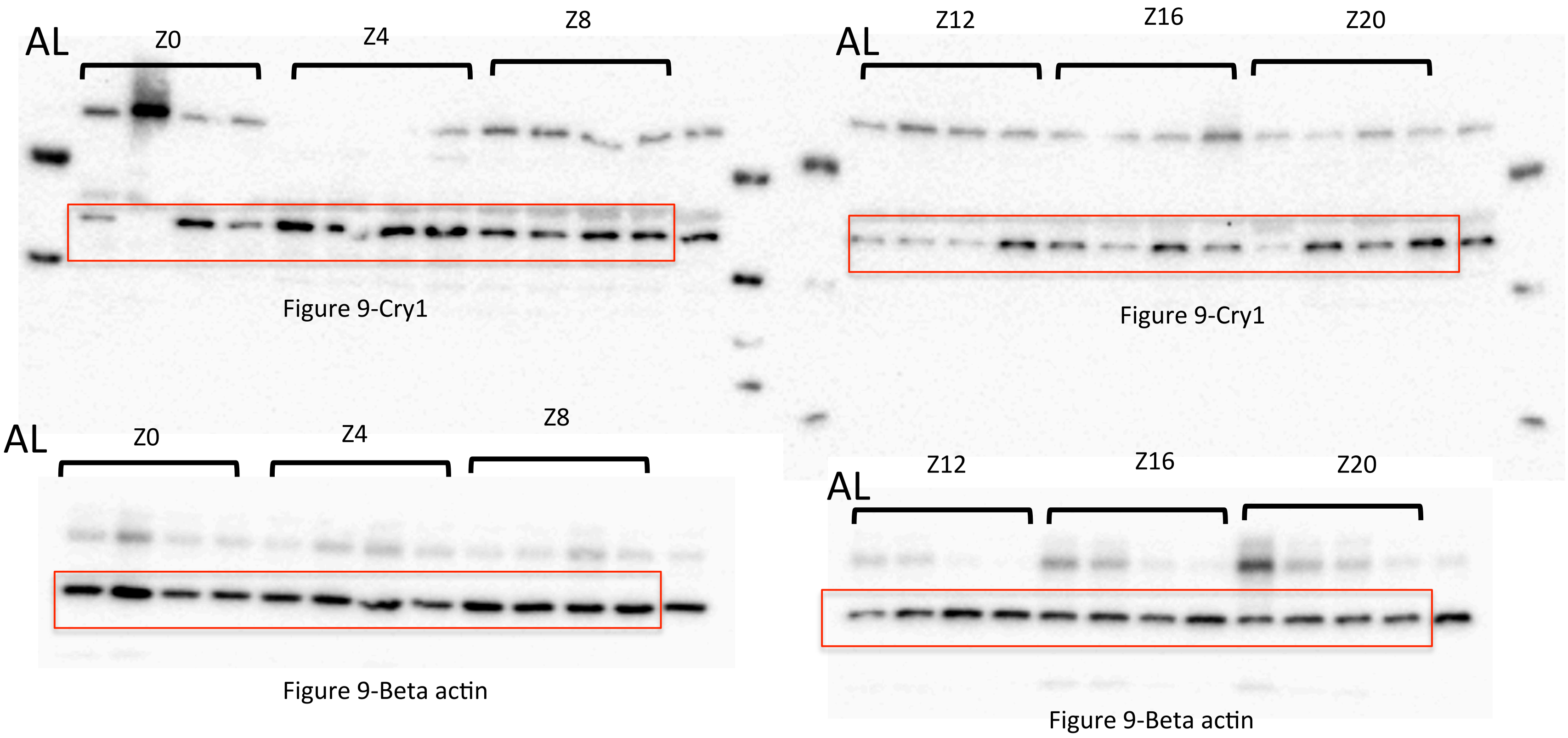
AL
Z0
Z4
Z8
Figure 9-Cry1
AL
Z12
Z16
Z20
Figure 9-Cry1
AL
Z0
Z4
Z8
Figure 9-Beta actin
AL
Z12
Z16
Z20
Figure 9-Beta actin

Figure 9c

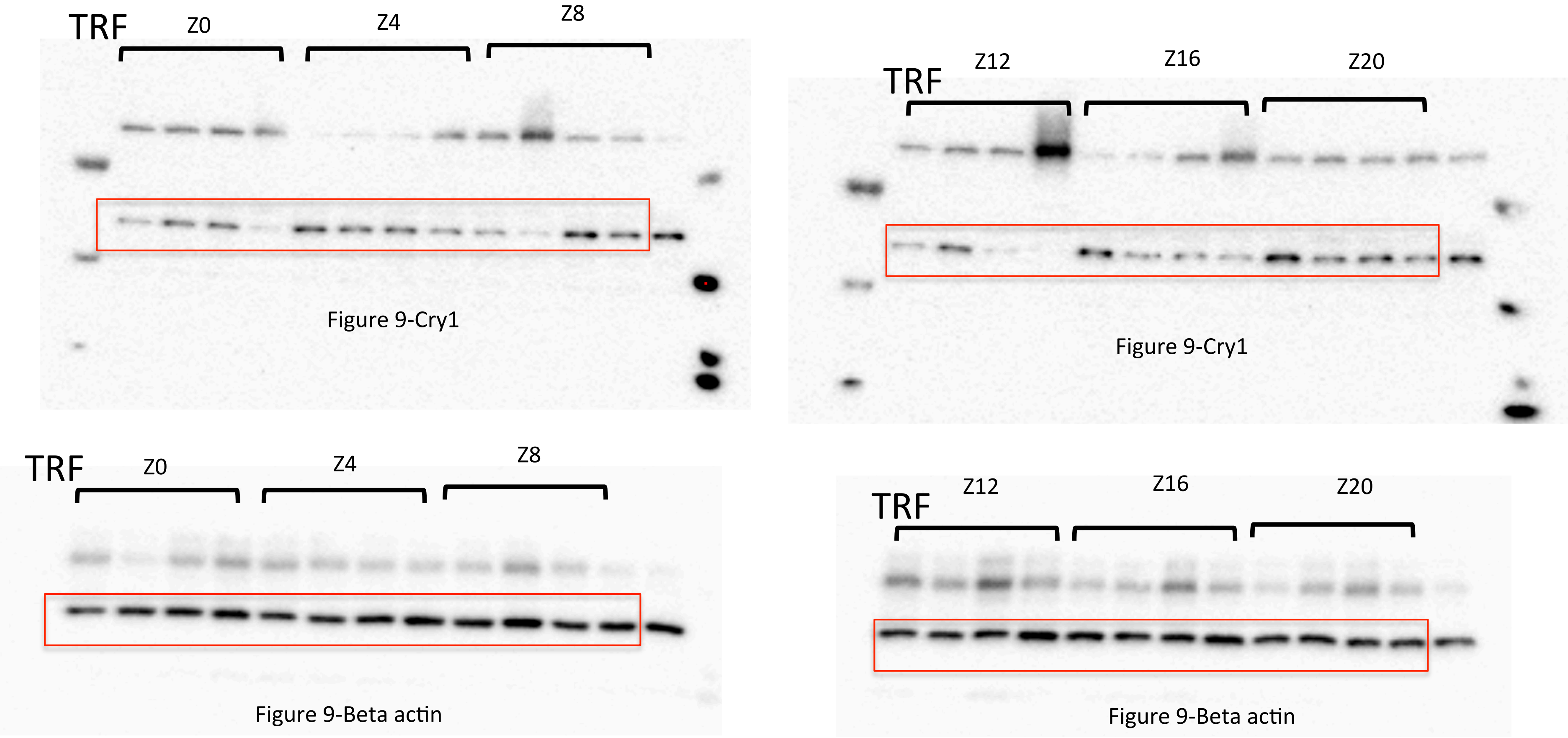
TRF
Z0
Z4
Z8
Figure 9-Cry1
TRF
Z12
Z16
Z20
Figure 9-Cry1
TRF
Z0
Z4
Z8
Figure 9-Beta actin
TRF
Z12
Z16
Z20
Figure 9-Beta actin

Figure 9c

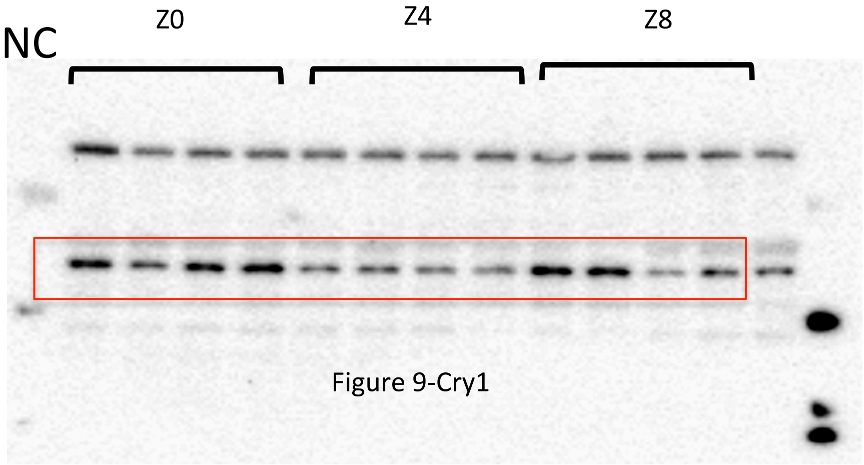

Figure 9-Cry1

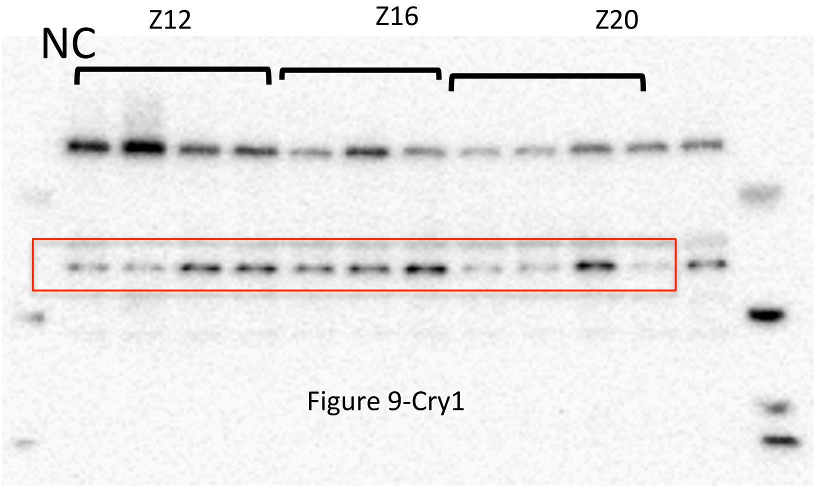

Figure 9-Cry1

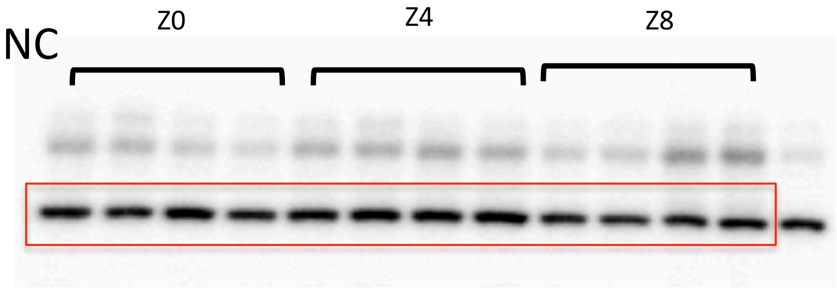

Figure 9-Beta actin

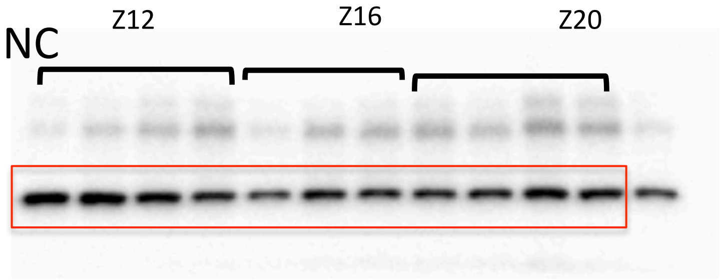

Figure 9-Beta actin

Figure 9e

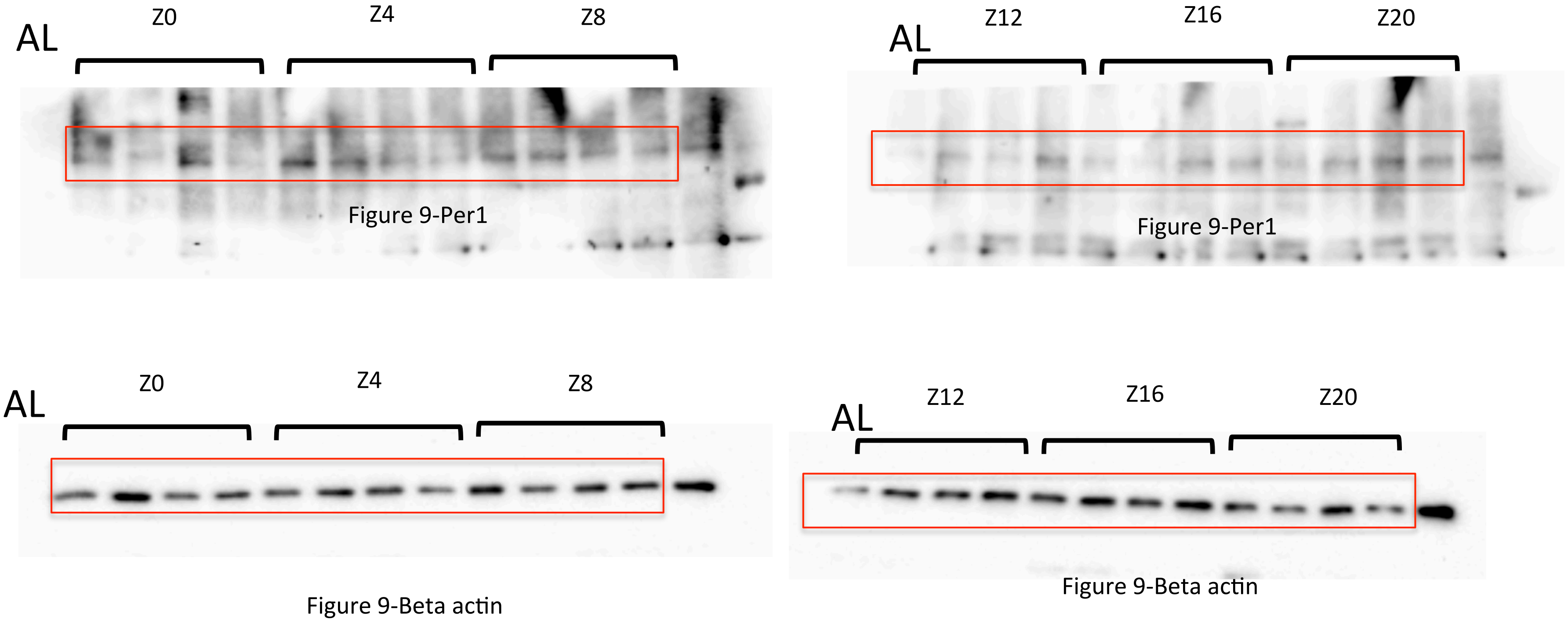
AL
Z0
Z4
Z8
Figure 9-Per1
AL
Z12
Z16
Z20
Figure 9-Per1
AL
Z0
Z4
Z8
Figure 9-Beta actin
AL
Z12
Z16
Z20
Figure 9-Beta actin

Figure 9e

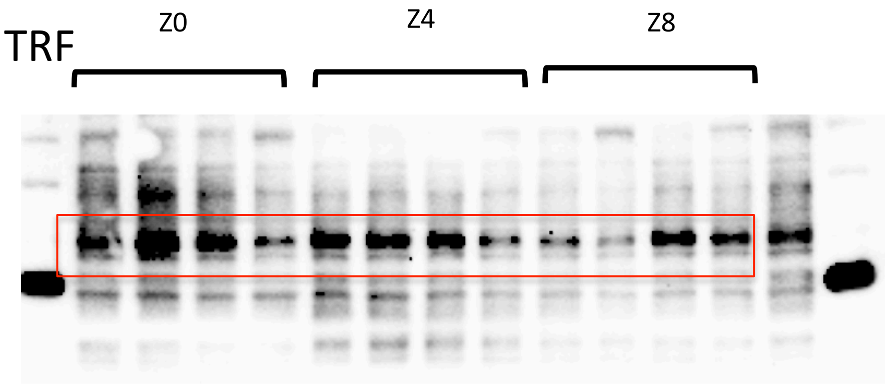

Figure 9-Per1

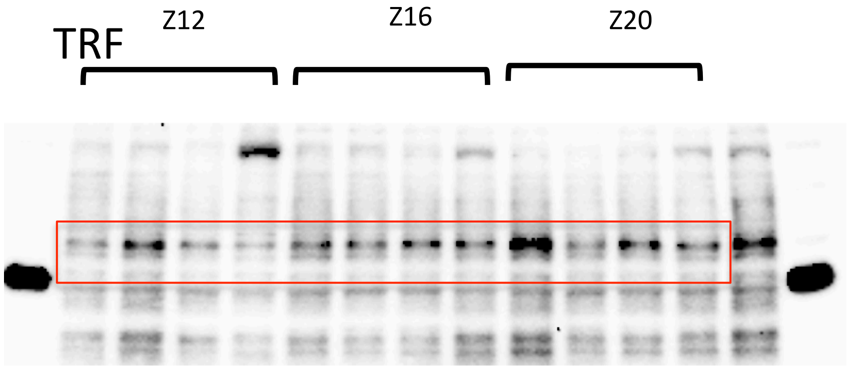

Figure 9-Per1

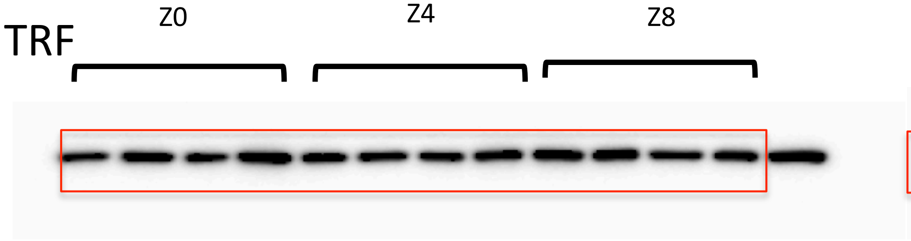

Figure 9-Beta actin

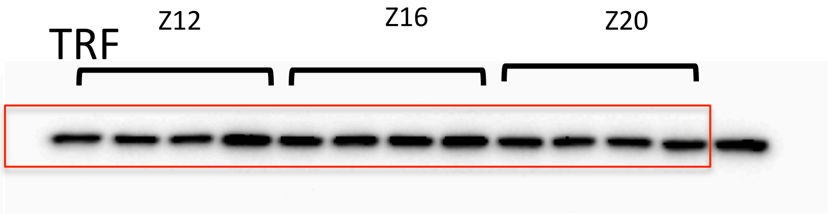

Figure 9-Beta actin

Figure 9e

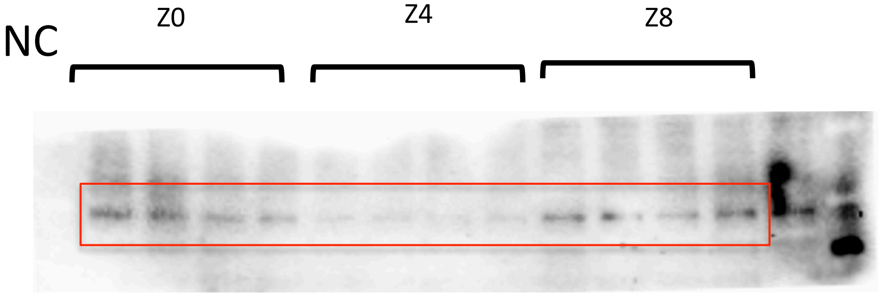

Figure 9-Per1

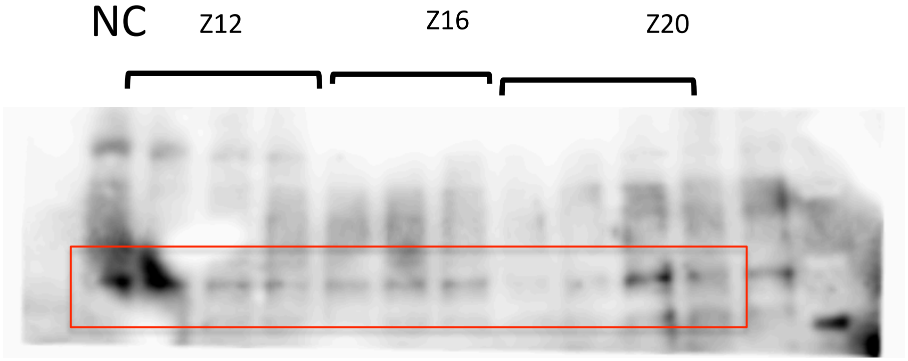

Figure 9-Per1

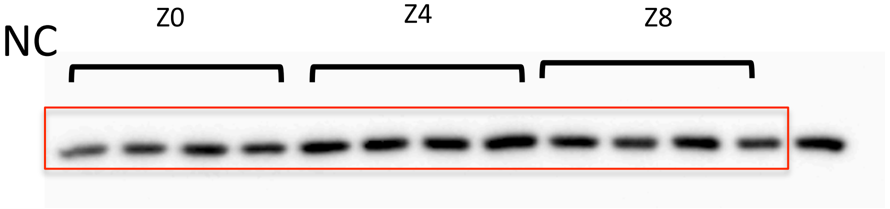

Figure 9-Beta actin

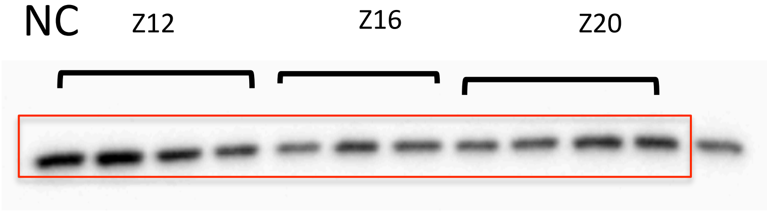

Figure 9-Beta actin

Figure S9a

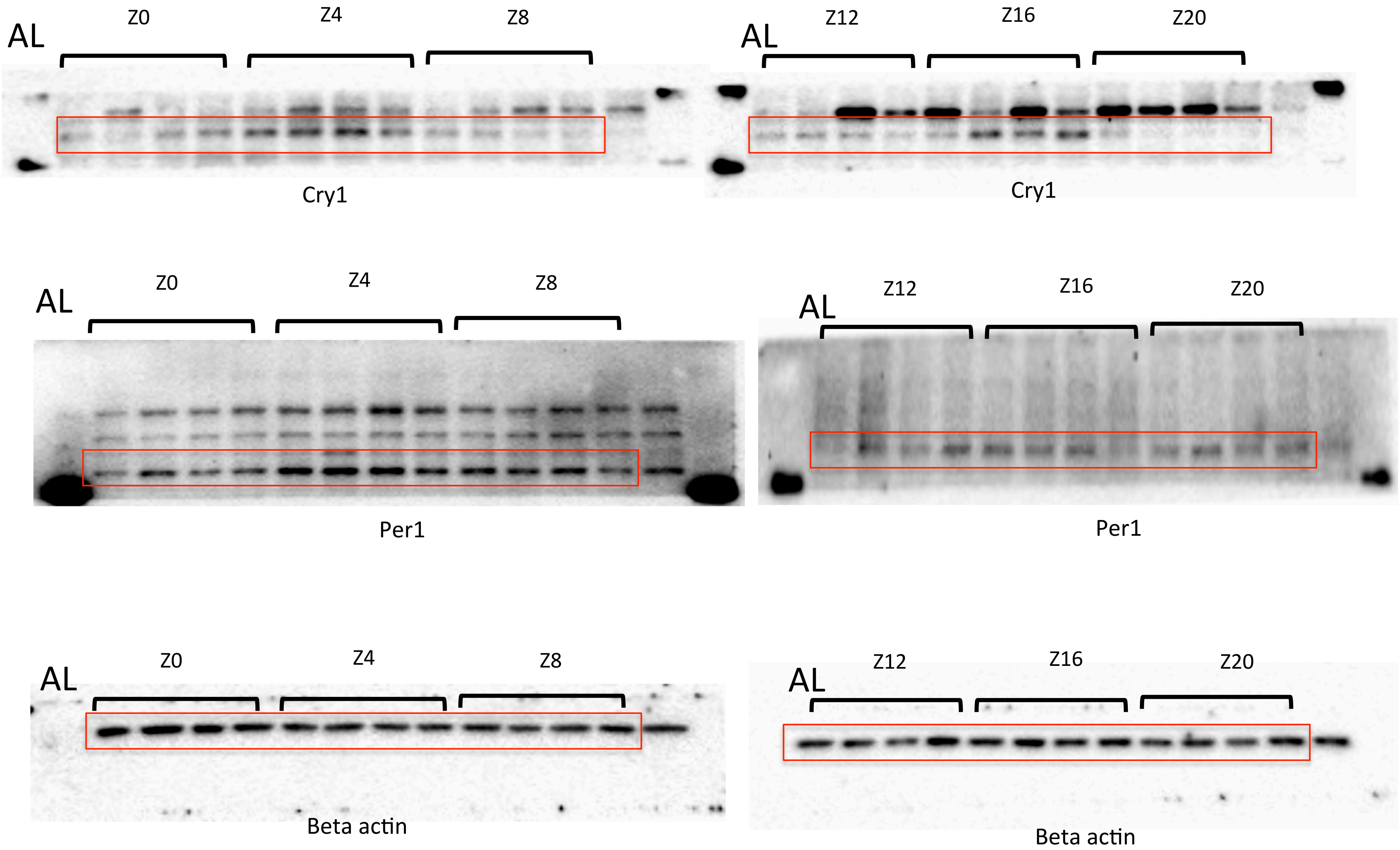
AL
Z0
Z4
Z8
Cry1
AL
Z12
Z16
Z20
Cry1
AL
Z0
Z4
Z8
Per1
AL
Z12
Z16
Z20
Per1
AL
Z0
Z4
Z8
Beta actin
AL
Z12
Z16
Z20
Beta actin

Figure S9a

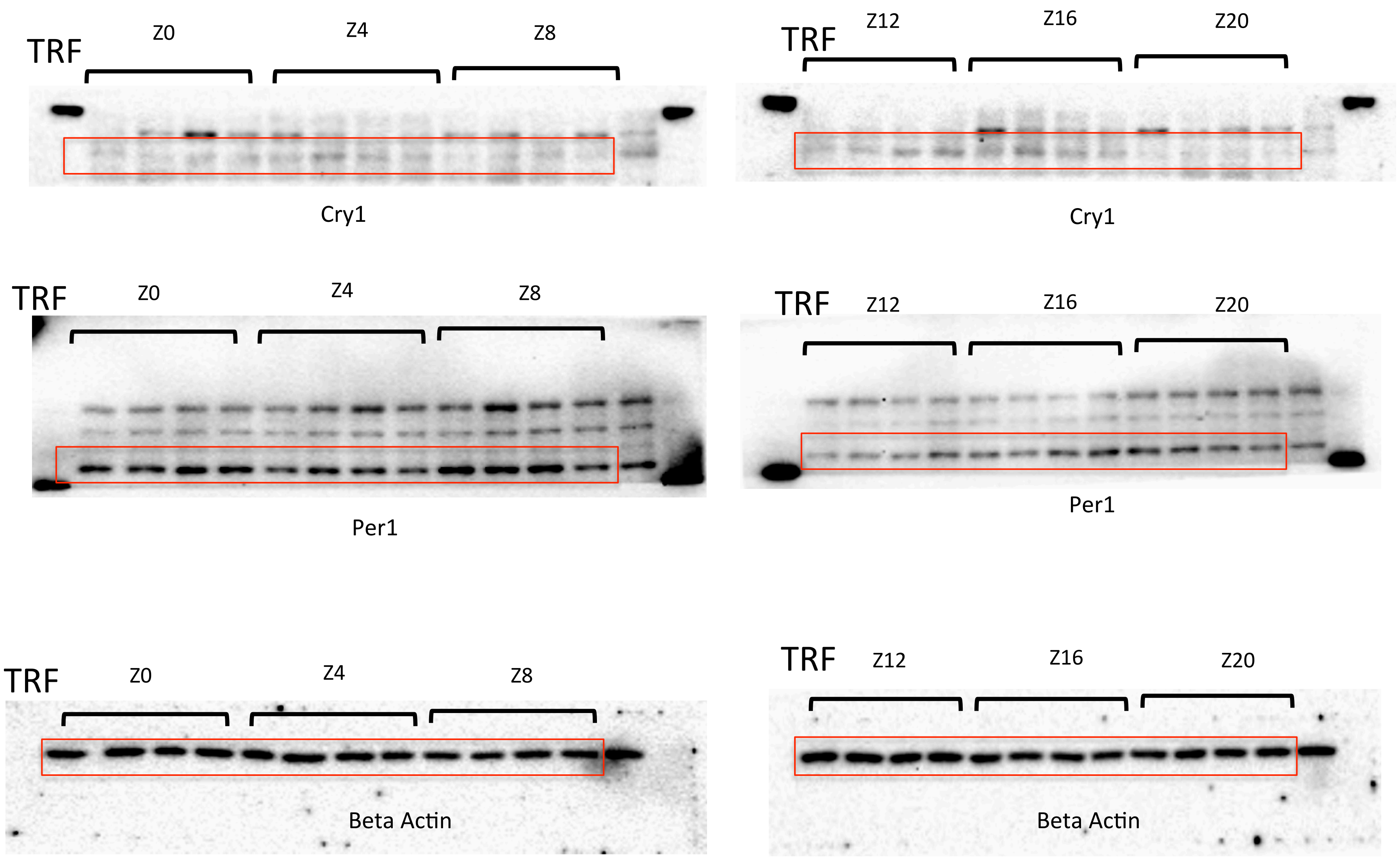
TRF
Z0
Z4
Z8
Cry1
TRF
Z12
Z16
Z20
Cry1
TRF
Z0
Z4
Z8
Per1
TRF
Z12
Z16
Z20
Per1
TRF
Z0
Z4
Z8
Beta Actin
TRF
Z12
Z16
Z20
Beta Actin

Figure S9a

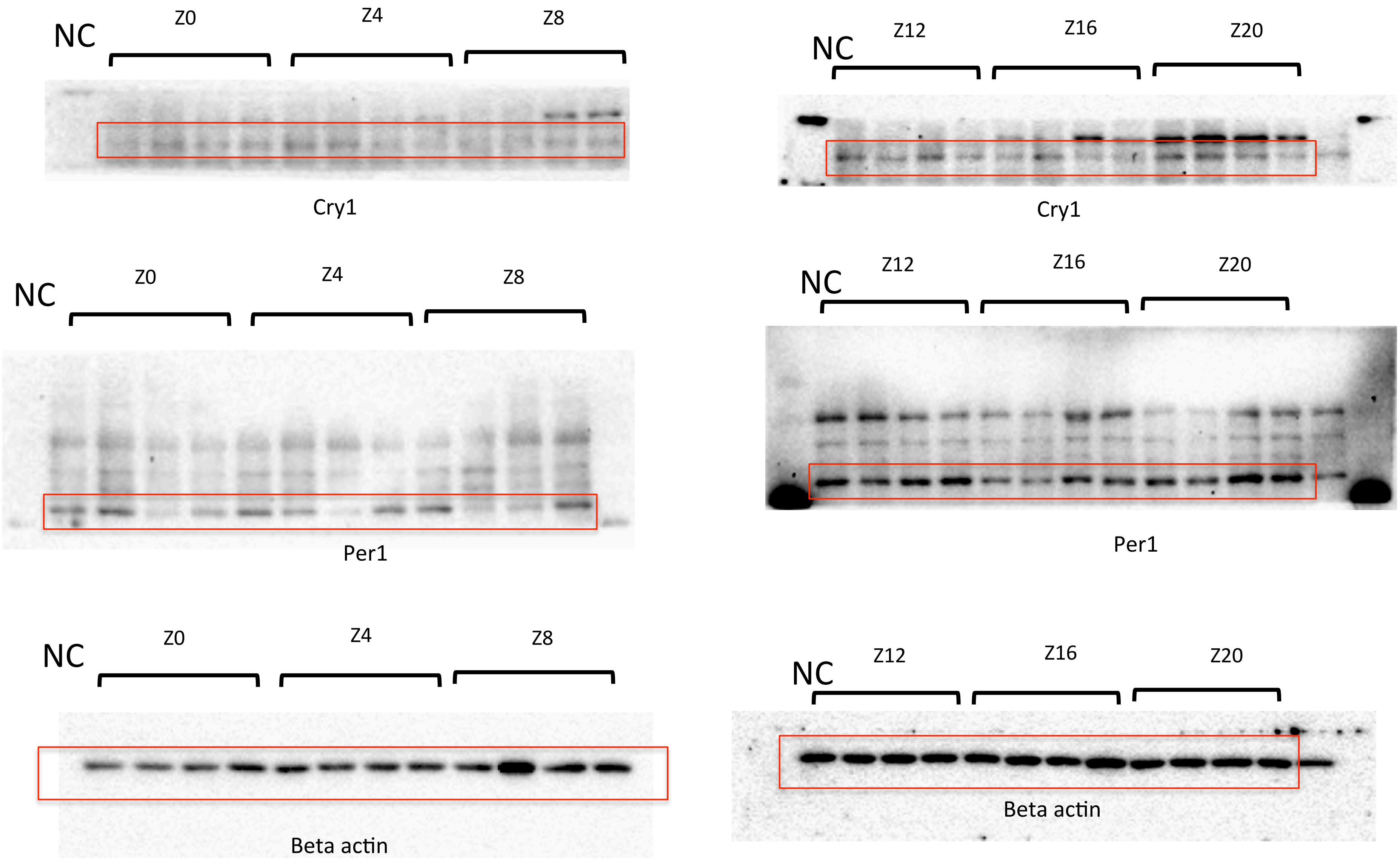

NC
Z0
Z4
Z8
Cry1
NC
Z12
Z16
Z20
Cry1
NC
Z0
Z4
Z8
Per1
NC
Z12
Z16
Z20
Per1
NC
Z0
Z4
Z8
Beta actin
NC
Z12
Z16
Z20
Beta actin

Figure S9d

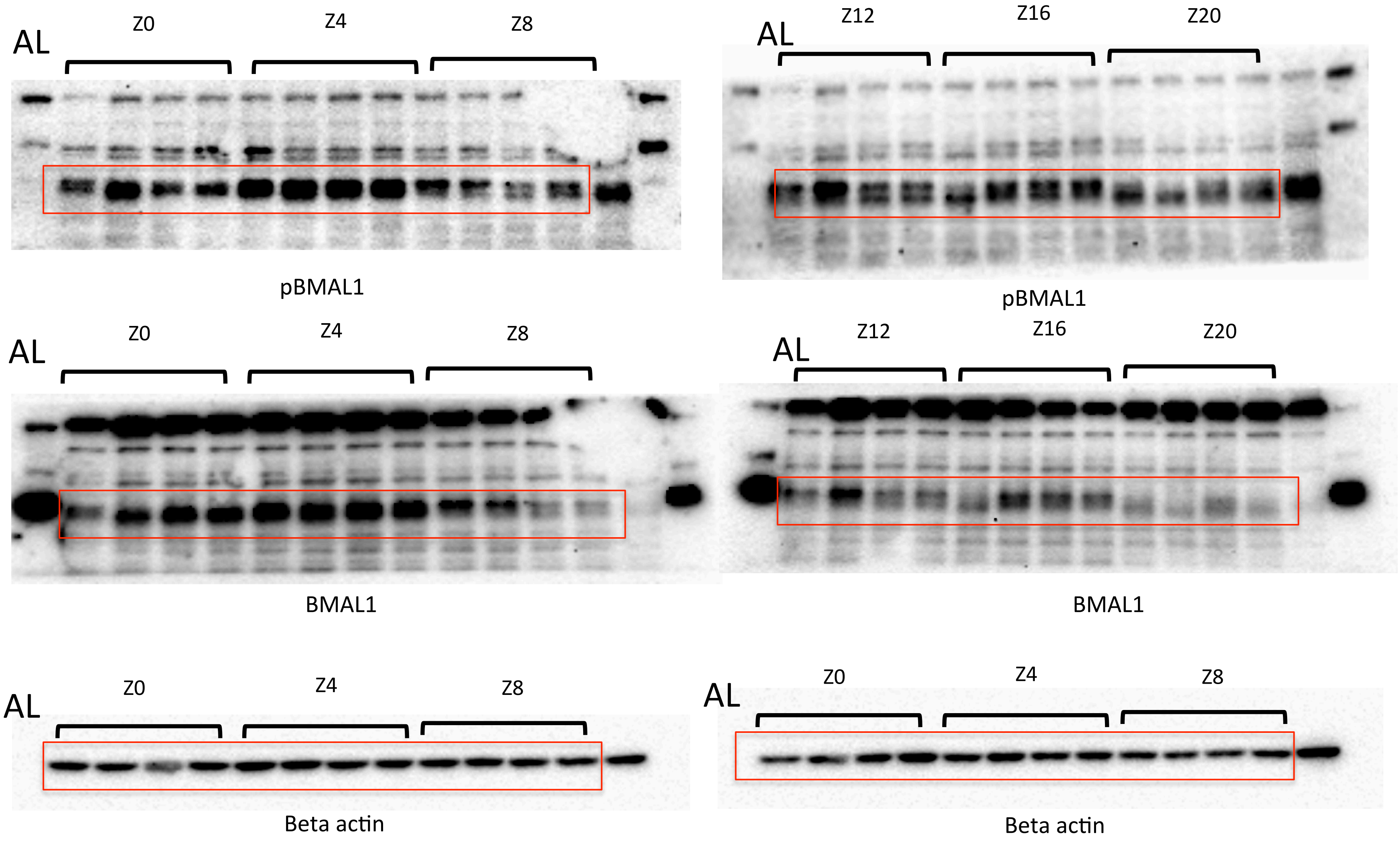
AL
Z0
Z4
Z8
pBMAL1
AL
Z12
Z16
Z20
pBMAL1
AL
Z0
Z4
Z8
BMAL1
AL
Z12
Z16
Z20
BMAL1
AL
Z0
Z4
Z8
Beta actin
AL
Z0
Z4
Z8
Beta actin

Figure S9d

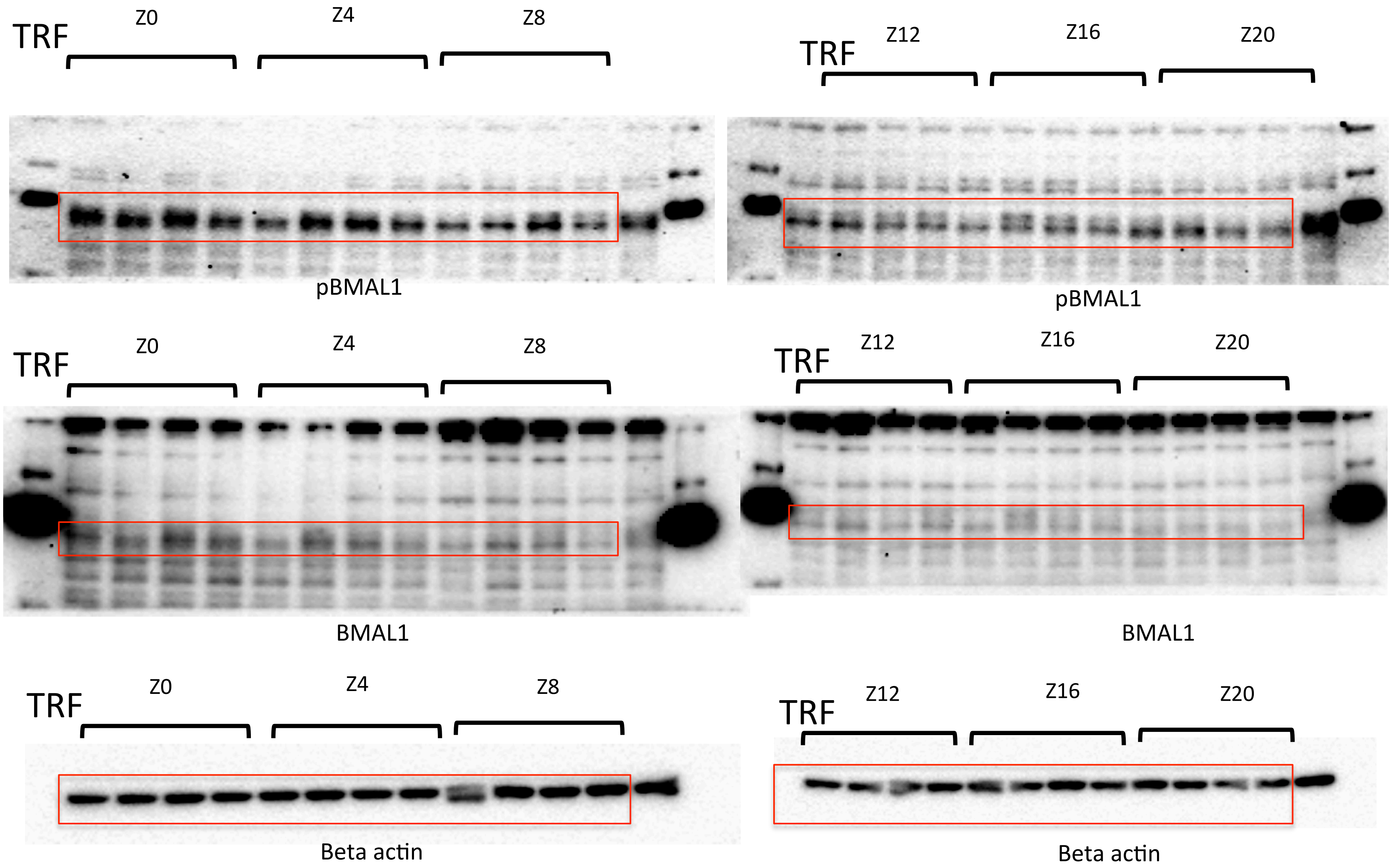
TRF
Z0
Z4
Z8
pBMAL1
TRF
Z12
Z16
Z20
pBMAL1
TRF
Z0
Z4
Z8
BMAL1
TRF
Z12
Z16
Z20
BMAL1
TRF
Z0
Z4
Z8
Beta actin
TRF
Z12
Z16
Z20
Beta actin

Figure S9d

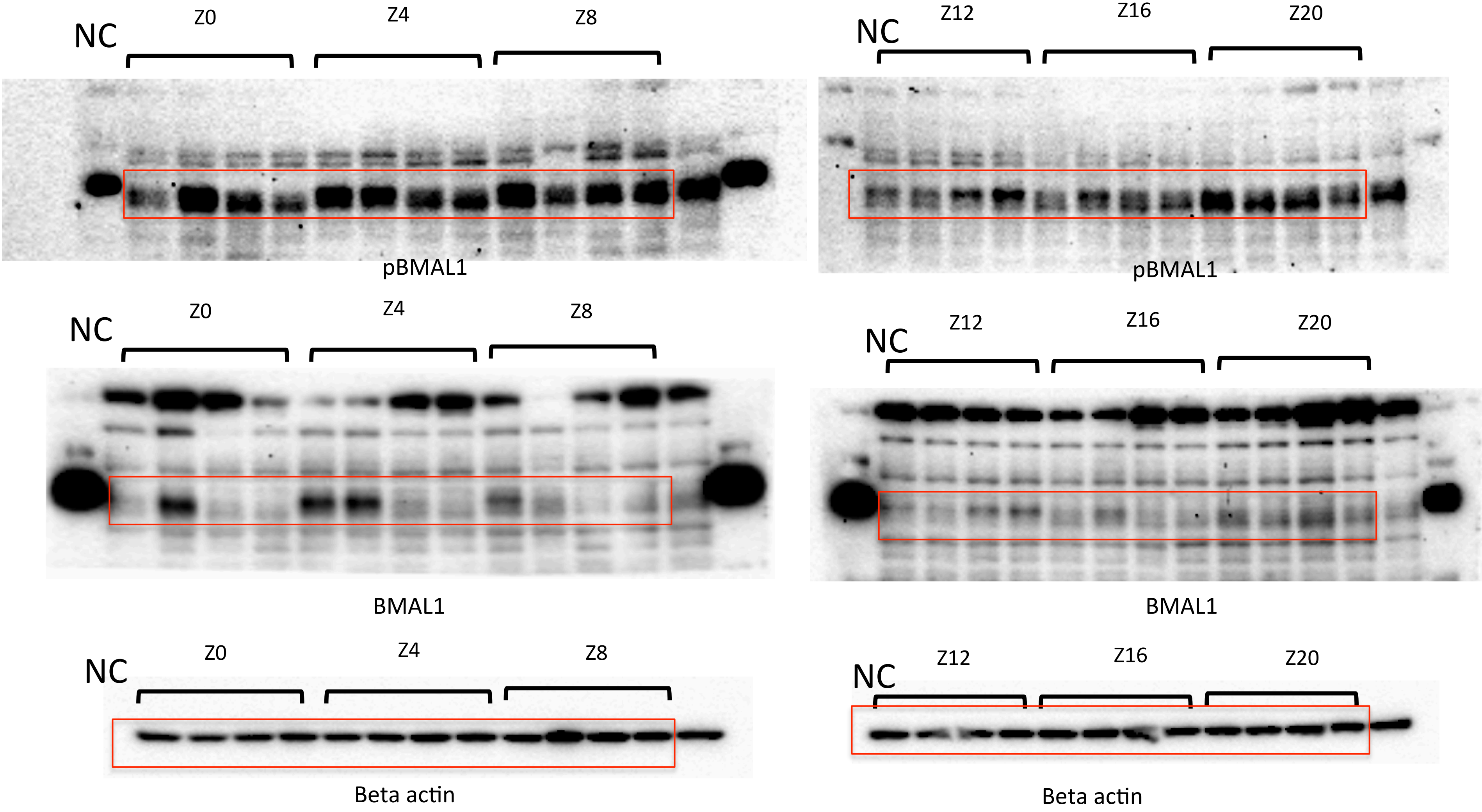
NC
Z0
Z4
Z8
pBMAL1
NC
Z12
Z16
Z20
pBMAL1
NC
Z0
Z4
Z8
BMAL1
NC
Z12
Z16
Z20
BMAL1
NC
Z0
Z4
Z8
Beta actin
NC
Z12
Z16
Z20
Beta actin

Figure S10a

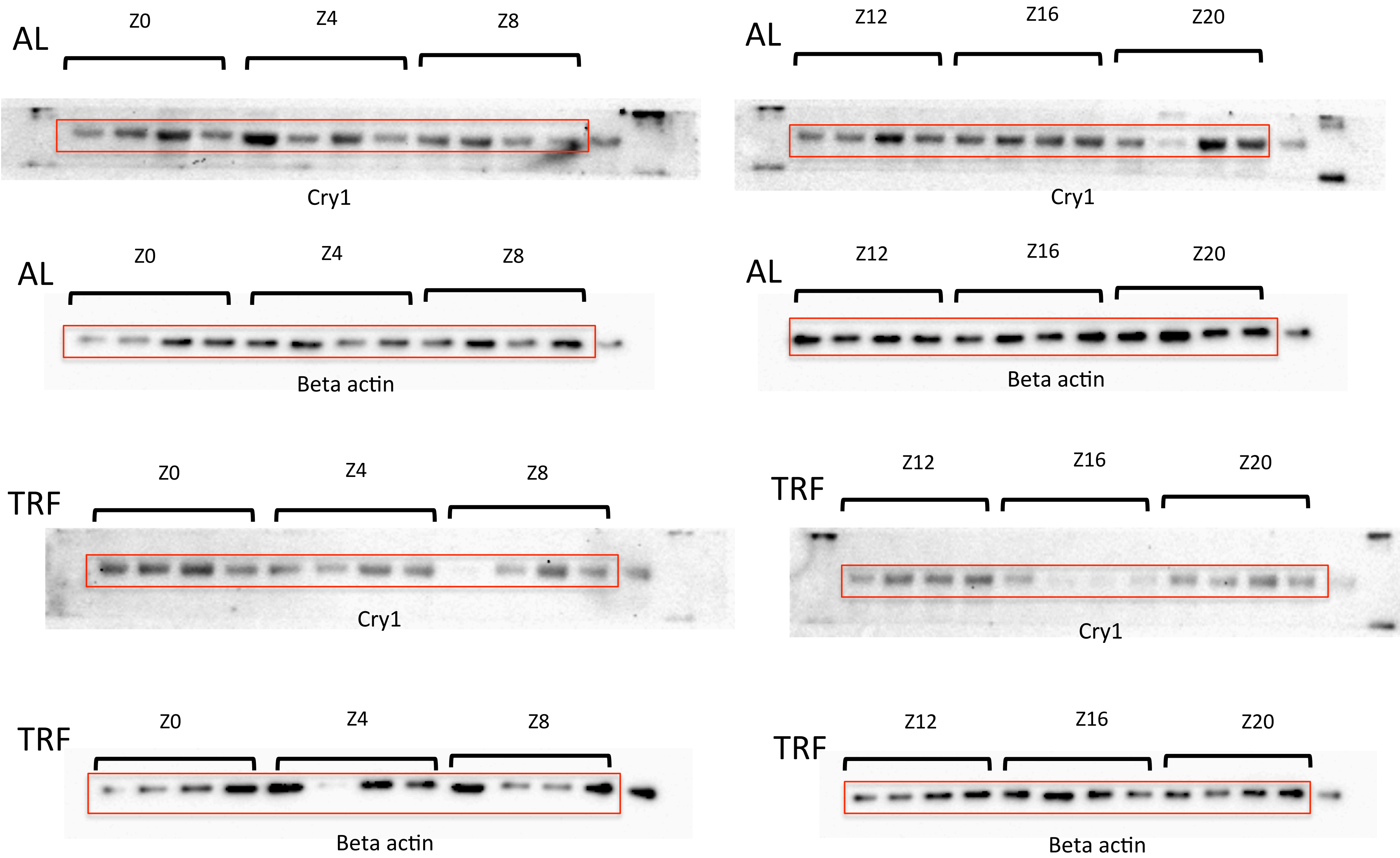
AL
Z0
Z4
Z8
Cry1
AL
Z12
Z16
Z20
Cry1
AL
Z0
Z4
Z8
Beta actin
AL
Z12
Z16
Z20
Beta actin
TRF
Z0
Z4
Z8
Cry1
TRF
Z12
Z16
Z20
Cry1
TRF
Z0
Z4
Z8
Beta actin
TRF
Z12
Z16
Z20
Beta actin

Figure S10a

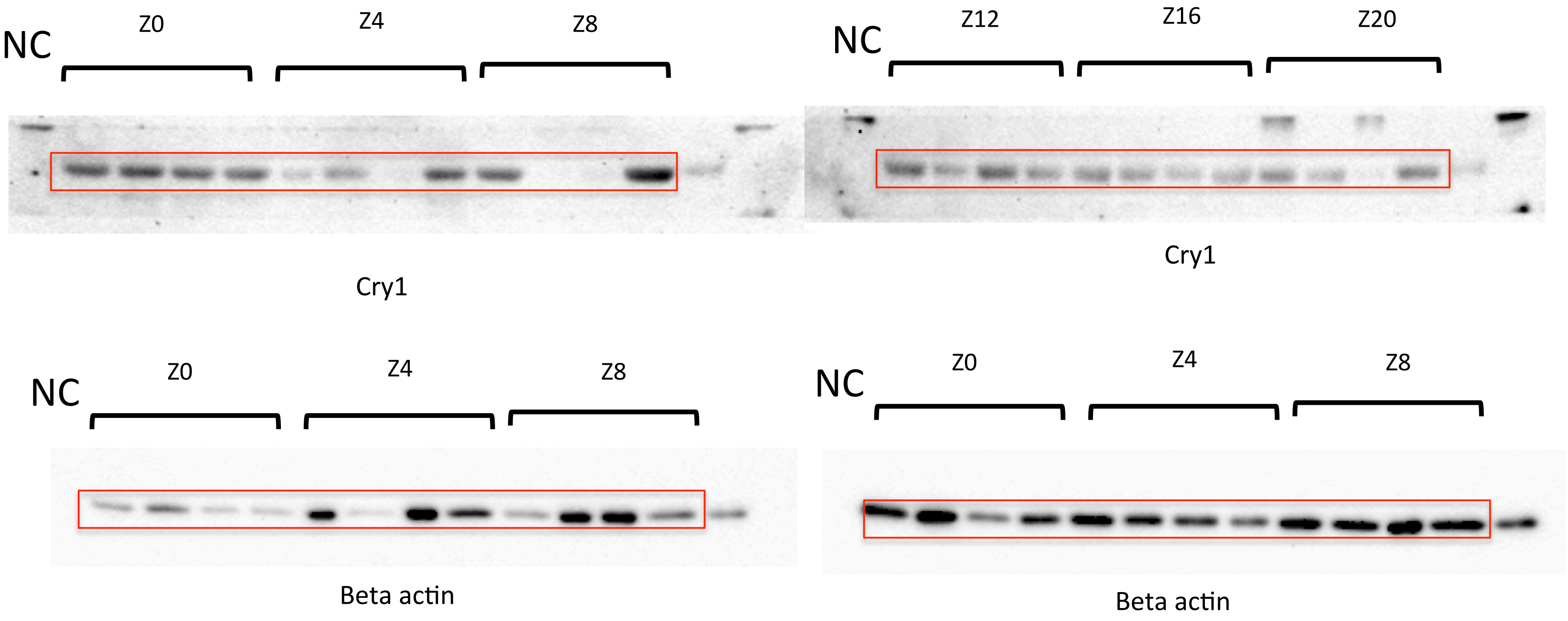
NC
Z0
Z4
Z8
Cry1
NC
Z12
Z16
Z20
Cry1
NC
Z0
Z4
Z8
Beta actin
NC
Z0
Z4
Z8
Beta actin

Figure S10c

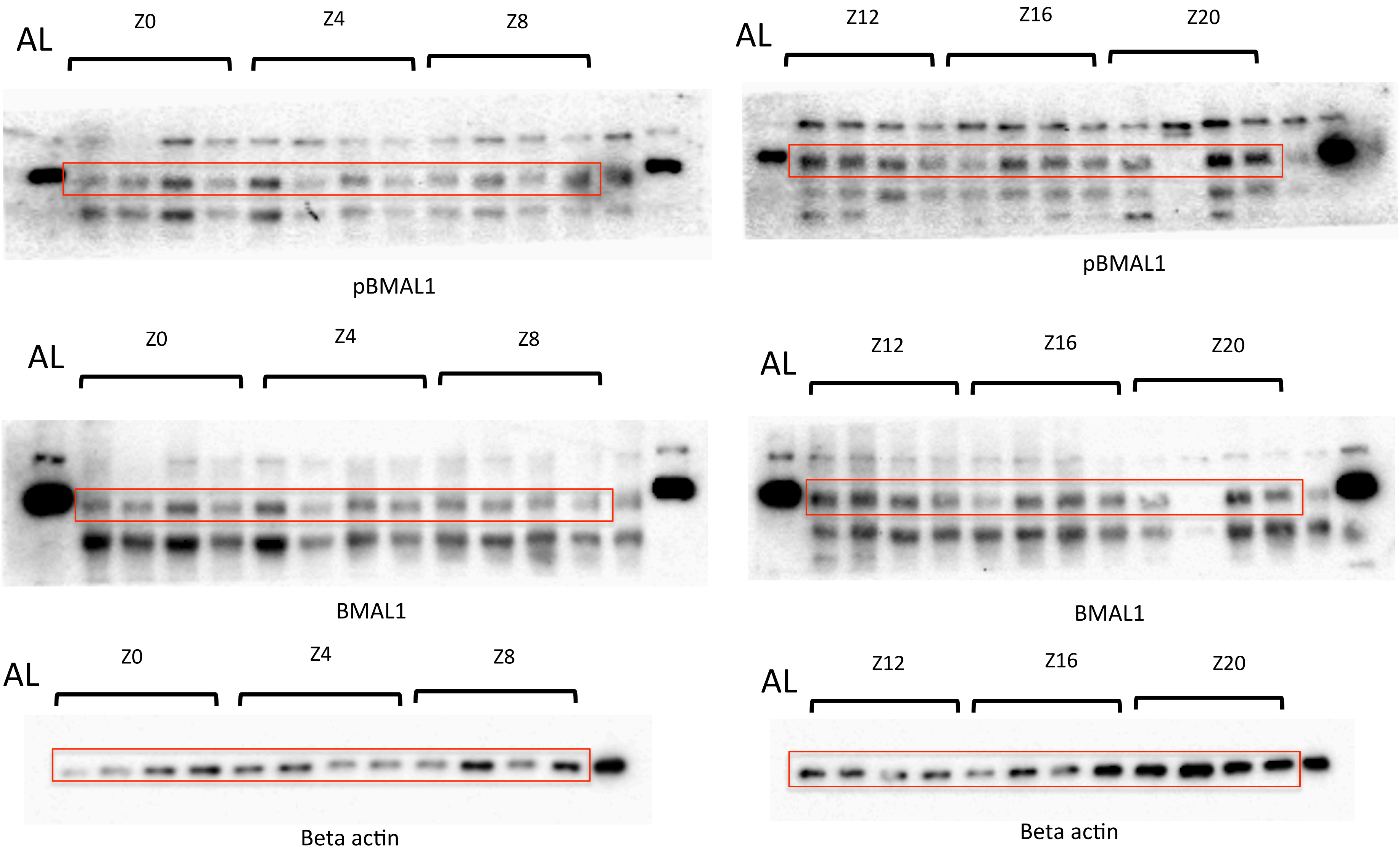
AL
Z0
Z4
Z8
pBMAL1
AL
Z12
Z16
Z20
pBMAL1
AL
Z0
Z4
Z8
BMAL1
AL
Z12
Z16
Z20
BMAL1
AL
Z0
Z4
Z8
Beta actin
AL
Z12
Z16
Z20
Beta actin

Figure S10c

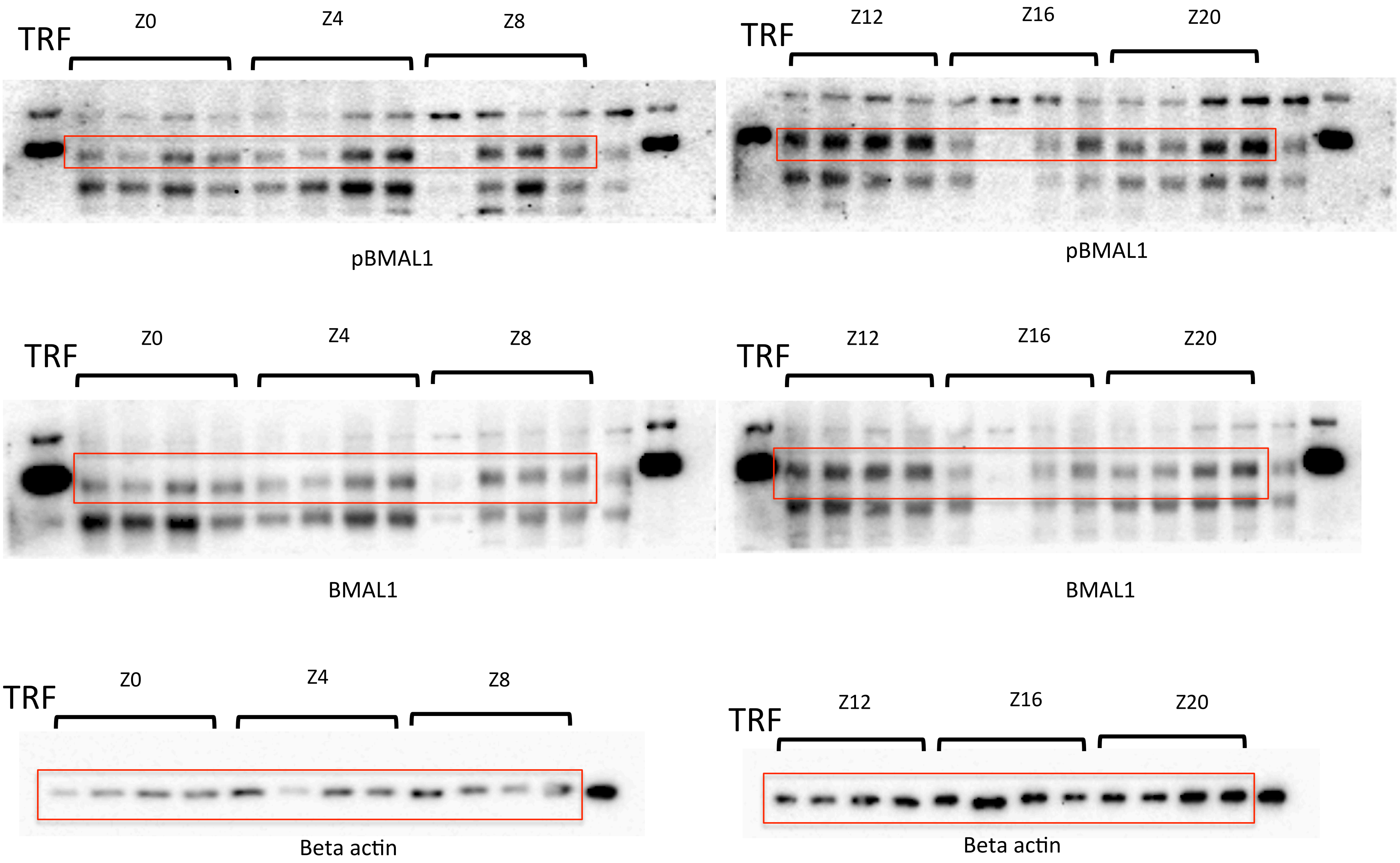
TRF
Z0
Z4
Z8
pBMAL1
TRF
Z12
Z16
Z20
pBMAL1
TRF
Z0
Z4
Z8
BMAL1
TRF
Z12
Z16
Z20
BMAL1
TRF
Z0
Z4
Z8
Beta actin
TRF
Z12
Z16
Z20
Beta actin

Figure S10c

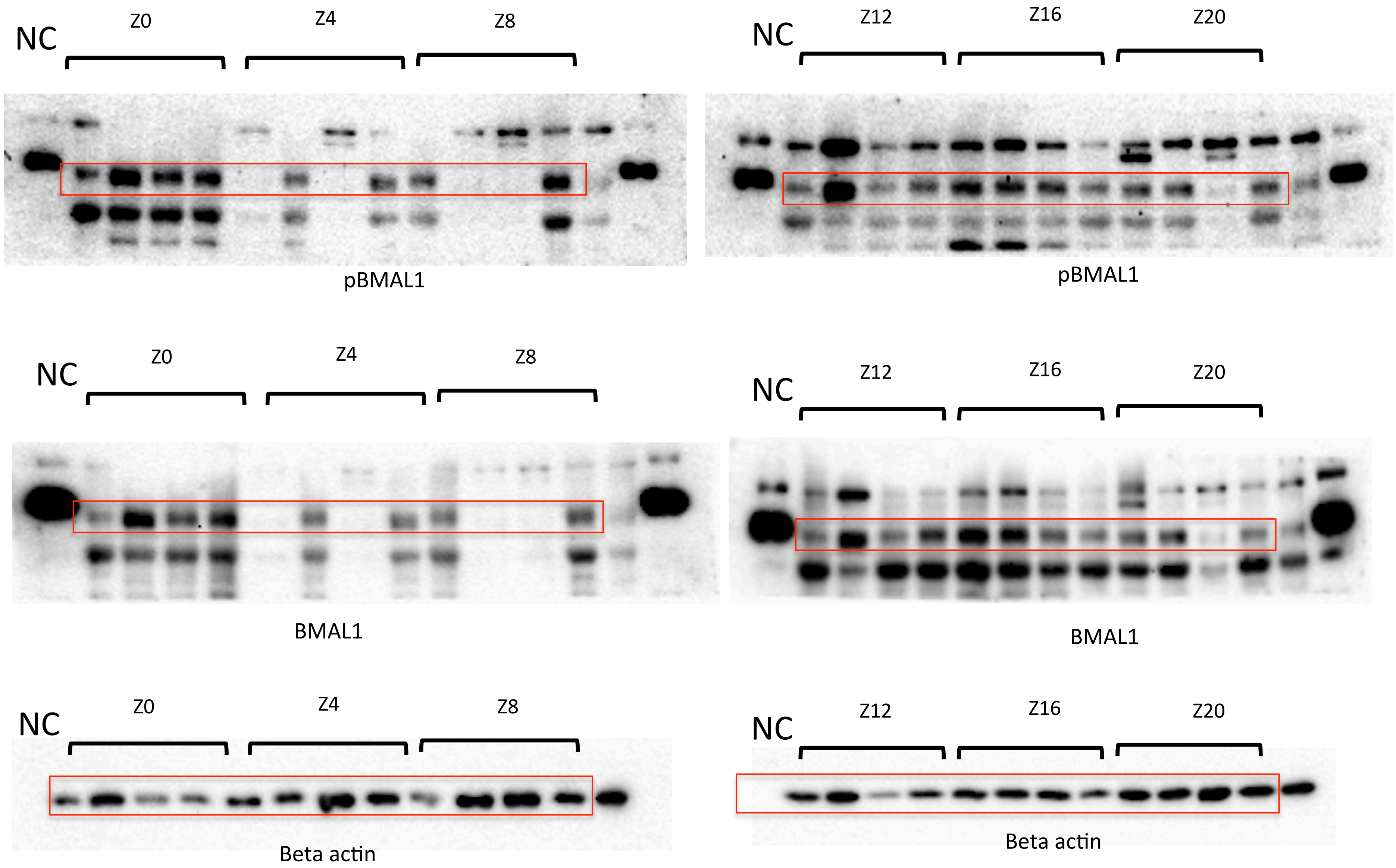
NC
Z0
Z4
Z8
pBMAL1
NC
Z12
Z16
Z20
pBMAL1
NC
Z0
Z4
Z8
BMAL1
NC
Z12
Z16
Z20
BMAL1
NC
Z0
Z4
Z8
Beta actin
NC
Z12
Z16
Z20
Beta actin
